# Dynamic Variation of Amino Acid Contents and Identification of Sterols in Xinyang Mao Jian Green Tea

**DOI:** 10.3390/molecules27113562

**Published:** 2022-06-01

**Authors:** Meng Sun, Fangfang Yang, Wanying Hou, Shuangfeng Jiang, Runqi Yang, Wei Zhang, Mingjie Chen, Yuhang Yan, Yuxin Tian, Hongyu Yuan

**Affiliations:** 1 Henan Key Laboratory of Tea Plant Biology, College of Life Sciences, Xinyang Normal University, Xinyang 464000, China; sunmeng2010abc@163.com (M.S.); battc9839bgrg0@163.com (W.H.); jshuangfeng@163.com (S.J.); yangrunqi246875@163.com (R.Y.); chawenhua2009@163.com (W.Z.); mjchen006@163.com (M.C.); 17839865339@163.com (Y.Y.); 15516796003@163.com (Y.T.); 2 Guangxi-ASEAN Food Inspection and Testing Center, Nanning 530029, China; yangfangfang18@126.com; 3 Xinyang Academy of Agricultural Sciences, Xinyang 464000, China

**Keywords:** Xinyang Mao Jian, amino acids, dynamic contents, sterols, growth process

## Abstract

As important biomolecules in *Camellia sinensis* L., amino acids (AAs) are considered to contribute to the overall green tea sensory quality and undergo dynamic changes during growth. However, limited by analytical capacity, detailed AAs composition in different growth stages remains unclear. To address this question, we analyzed the dynamic changes of 23 AAs during leaf growth in Xinyang Mao Jian (XYMJ) green tea. Using amino acid analyzer, we demonstrated that most AAs are abundant on Pure Brightness Day and Grain Rain Day. After Grain Rain, 23 AAs decreased significantly. Further analysis shows that theanine has a high level on the day before Spring Equinox and Grain Rain, accounting for 44–61% of the total free AAs content in tea leaves. Glu, Pro, and Asp are the second most abundant AAs. Additionally, spinasterol and 22,23-dihydrospinasterol are first purified and identified in ethanol extract of XYMJ by silica gel column chromatography method. This study reveals the relationship between plucking days and the dynamic changes of AAs during the growth stage and proves the rationality of the traditional plucking days of XYMJ green tea.

## 1. Introduction

The dynamic changes of amino acids (AAs) in tea plants during growth influence the overall taste of green tea infusions [1]. AAs not only contribute to the different taste characteristics of green tea infusions, but also provide precursors for the development of tea aroma [2,3,4]. Therefore, the contents of AAs play an important role in tea quality. Understanding the variation in AAs contents during growth will provide insights into the relationship between the harvest time and green tea quality. However, AAs contents in different growth process involve both increasing contents attributed to protein break-down, hydrolysis, or cleavage and decreasing content due to their consumption as precursors of other compounds [5,6]. The dynamic changes are detrimental for determining the reasonable plucking seasons, especially for Xinyang Mao Jian (XYMJ), whose annual average temperature is lower than that of other green tea-producing areas in China [7]. The special environmental condition has a significant effect on the contents of AAs and the formation of tea quality. Although many studies have focused on the changing of AAs during the processing technology, the dynamic changes of AAs in XYMJ green tea during growth processes have not been systematically studied.

Bioactive phenolic compounds, amino acids, organic acids, alkaloids, and sugars comprise the organoleptic characteristics of green tea infusions. Among them, amino acids are crucial to the taste and flavor attributes of tea. Free AAs can be divided into sweet (Gly, Ser, Ala, Pro, Thr, Trp, Cys, and Met), bitter (Arg, His, Ile, Leu, Phe, Lys, Tyr, and Val), and umami (Asp, Glu, and Thea) AAs according to their taste characteristics [8,9]. The brothy sweet umami taste note of the green tea is due to umami AAs. Moreover, AAs impart numerous biological benefits, including anti-inflammatory, antimicrobial, anti-oxidant, and neurological effects. Additionally, some AAs even have positive and significant correlation with other sensory quality (including aroma, appearance, liquor, and total quality) and provide precursors for the development of tea aroma and flavor. For example, Thea takes part in the biosynthesis of polyphenols. Thus, detection of AAs in green tea is of great significance. Notably, tea cultivars have a high economic value due to their high AA concentration, especially for umami AAs. Traditionally, the plucking season of XYMJ green tea always begins before Pure Brightness and ends with Grain Rain. Thousands of years of traditional experience shows that XYMJ green tea plucked before Pure Brightness delivers higher quality than that obtained from other seasons. However, to date, scanty information is available on the dynamic changes of AAs components during new shoot development in these cultivars caused by growth stages, although the prices consumers pay depend on it.

In general, spectroscopic methods, capillary electrophoresis, chromatographic methods, and electro-chemical methods have been developed for investigating AA variation during the manufacturing process stage [10,11,12,13,14]. In order to clarify the relationship between the withering process and green tea quality, dynamic changes of AAs in green tea were investigated by Tong et al. [15]. They found that Thea content decreased slightly with fluctuations, Glu and Asp contents presented an increase–decrease pattern, whereas Pro contents remained almost constant. Xu found that the Thea concentration in the yellow cultivar was significantly higher than that in the green cultivar, and increased gradually as the leaves matured until they reached a maximum in the one bud and three leaves stage [16]. Although the above studies focus on the dynamic changes of main AAs, the dynamic changes of 23 AAs in XYMJ during the growth stage has barely been reported, which would be disadvantageous for XYMJ tea with non-constant growing conditions throughout the year, since the relationship between growth process and green tea quality cannot be clearly known. In addition, AAs, flavonoids, phenolic acids, purine alkaloids, catechins, and caffeine are highly related to green tea [17,18,19], it is unclear whether other types of compounds exist in XYMJ green tea. Chen et al. demonstrated that steroids and triterpenes are the main compounds present in tea leaves [20]. Until now, the contents of AAs contributing to quality characteristics in XYMJ related to tea growth and material foundation are still uncertain.

In this paper, a sensitive method for the quantitative detection of 23 AAs in XYMJ green tea during the growth stages has been developed by using a Hitachi AA analyzer. We showed that 23 AAs present a dynamic change (decrease-increase-decrease-increase-decrease pattern) from the day before Spring Equinox to nine days after Grain Rain. Contents of 23 AAs appeared two peaks on Pure Brightness Day and Grain Rain Day. Thea was the most abundant AA in XYMJ green tea, accounting for 44–61% of the total free AAs content in tea leaves from the day before Spring Equinox to Grain Rain. Glu, Pro, and Asp were the other major AAs. Contents of Gln, Asn, Arg, Ser, Phe, Thr, Tyr, Lys, and Leu were in the third gradient. Moreover, spinasterol and 22,23-dihydrospinasterol were first isolated and identified from XYMJ green tea by silica gel column chromatography. Our study reveals the relationship between plucking days and the dynamic changes of AAs during the growth stage and proved the rationality of the traditional plucking days (starting before Pure Brightness and ending with Grain Rain) of XYMJ green tea.

## 2. Results

### 2.1. Structures Elucidation of Spinasterol and 22,23-Dihydrospinasterol

**Figure 1:**
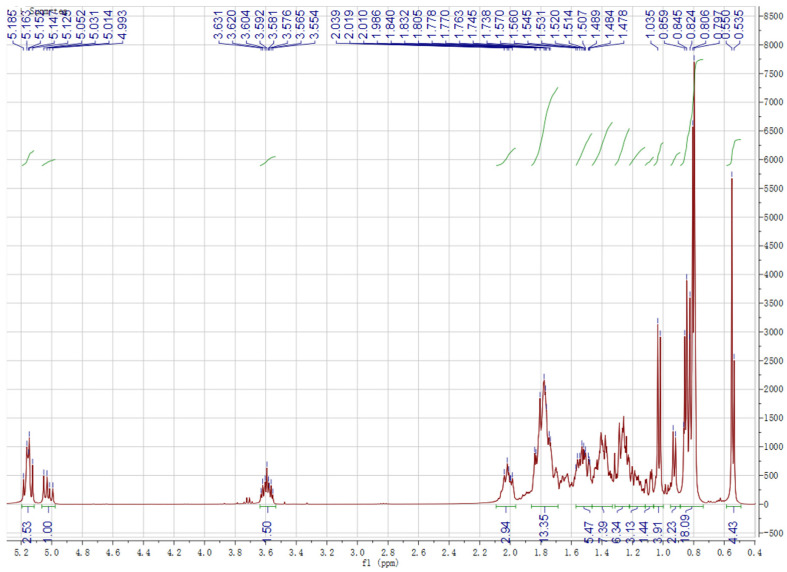
^1^H NMR spectrum of the obtained compounds.

**Figure 2:**
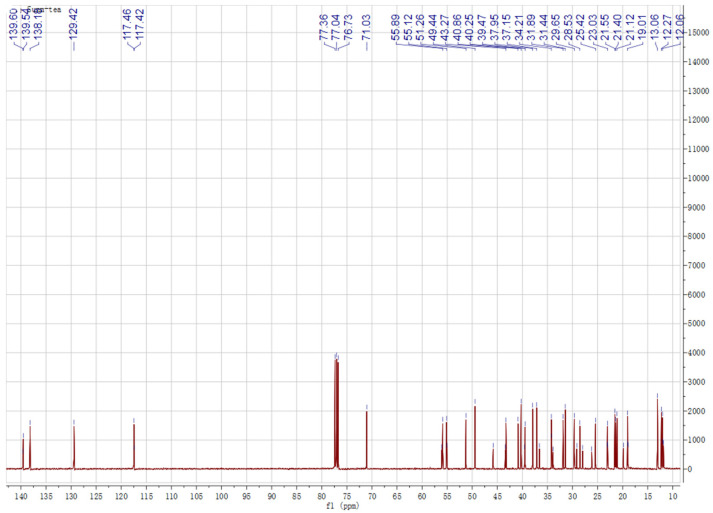
^13^C NMR spectrum of the obtained compounds.

Chemical structures of the identified compounds obtained from silica gel column chromatography in experimental sections were characterized by ^1^H-NMR and ^13^C-NMR spectroscopy (Figure 1 and Figure 2). Analysis of the ^1^H and ^13^C NMR spectra of spinasterol revealed 29 carbons signals comprising six methyls (11.99, 12.27, 13.06, 18.92, 21.40, 21.12) and two double bonds (117.40, 139.54; 129.42, 138.18). An oxygen substituted CH was constructed from the H signals *δ*_H_ 3.60 (*δ*_C_ 71.03, H-3) and two protons signals (*δ*_H_ 5.16, overlapped; 5.04, dd, *J* = 15.1, 8.8 Hz) were assignable to *cis*-oriented double bond (C22 and C23). These results suggest that it is an ergosterol. Considering that the ^13^C NMR spectrum was in generally good agreement with the known spinasterol, thus the isolated compounds contained spinasterol. In the same way, another compound was identified as 22,23-dihydrospinasterol [21]. Although the purified compound was a little bit impure, mixture of these two compounds was identified as a ratio of 1:2 with the comparison of the integral areas of the double bond region. Thus, spinasterol and 22,23-dihydrospinasterol were first isolated from XYMJ and identified by our experiment.

### 2.2. Decrease-Increase-Decrease-Increase-Decrease Pattern of AAs Contents

The taste and aroma that is attributed to tea quality, depend not only on the manufacturing method and geographical difference but also on the growth seasons of fresh tea shoots used as a raw material for processing [3]. Even if the tea shoots were grown in the same place and manufactured under the same conditions, the tea products have different market prices based on the growth stage of fresh tea shoots, which has been the major factor that determines green tea quality. In the case of the XYMJ green tea market, the tea plucked and tea product processed before Pure Brightness are believed to be of highest quality, thus the most famous and expensive type, followed by those are tea plucked on Grain Rain day, tea plucked on days after Grain Rain exhibits the lowest economic value. However, the dynamic changes of amino acids during the above growth period are not known.

**Figure 3:**
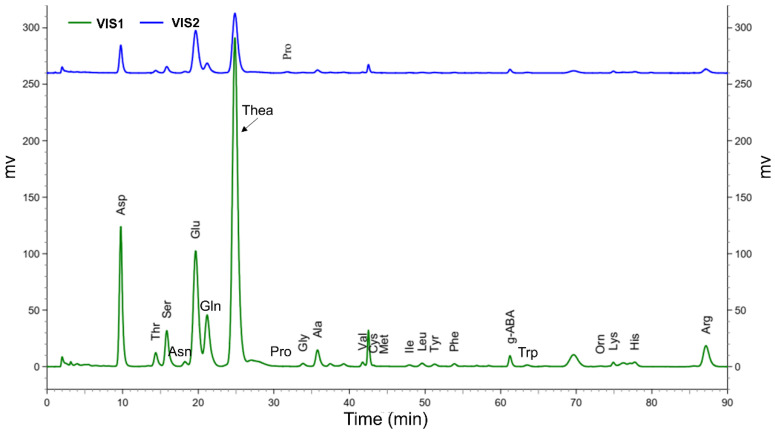
Hitachi AA analyzer report of 23 free amino acids: VIS 1 (green line) and tea samples VIS 2 (blue line). The retention time of Asp, Thr, Ser, Asn, Glu, Gln, Thea, Pro, Gly, Ala, Val, Cys, Met, Ile, Leu, Tyr, Phe, g-ABA, Trp, Orn, Lys, His, and Arg were 9.753, 14.38, 15.833, 18.253, 19.66, 21.18, 24.853, 31.747, 33.86, 35.78, 41.74, 43.147, 44.567, 47.94, 49.613, 51.267, 53.887, 61.24, 63.5, 73.213, 74.9, 77.747, and 87.16 min, respectively.

Due to the endogenous proteolytic enzymes and oxidases in XYMJ plucked at different growth periods are distinct, it will result in the diverse water-soluble proteins that can be hydrolyzed into free AAs by peptidase. This leads to a diversified content of 23 AAs in different XYMJ growing periods that produces its unique taste. In this study, considering the same cultivated place, manufacturing process, and conditions of buds and leaves, the dynamic changes of 23 AAs in XYMJ green tea during growth stages have been developed by using a Hitachi AA analyzer. The gradient and pH value of the elution buffer, which were observed to strongly affect the separation potency, were developed to achieve a sufficient separation resolution for all detected amino acids, especially for theanine, with the most abundant peak in the chromatogram (Figure 3). Peaks in tea infusion were identified by comparing with retention index including retention times of the authentic standards. As shown in Figure 3, the retention time of Asp, Thr, Ser, Glu, Gly, Ala, Val, Cys, Met, Ile, Leu, Tyr, Phe, g-ABA, Orn, Lys, His, Arg, Pro, Asn, Gln, Trp, and Thea were 9.753, 14.38, 15.833, 19.66, 33.86, 35.78, 41.74, 43.147, 44.567, 47.94, 49.613, 51.267, 53.887, 61.24, 73.213, 74.9, 77.747, 87.16, 31.747, 18.253, 21.18, 63.5, and 24.853 min, respectively.

### 2.3. Pure Brightness and Grain Rain Are the Key Growth Process

**Figure 4:**
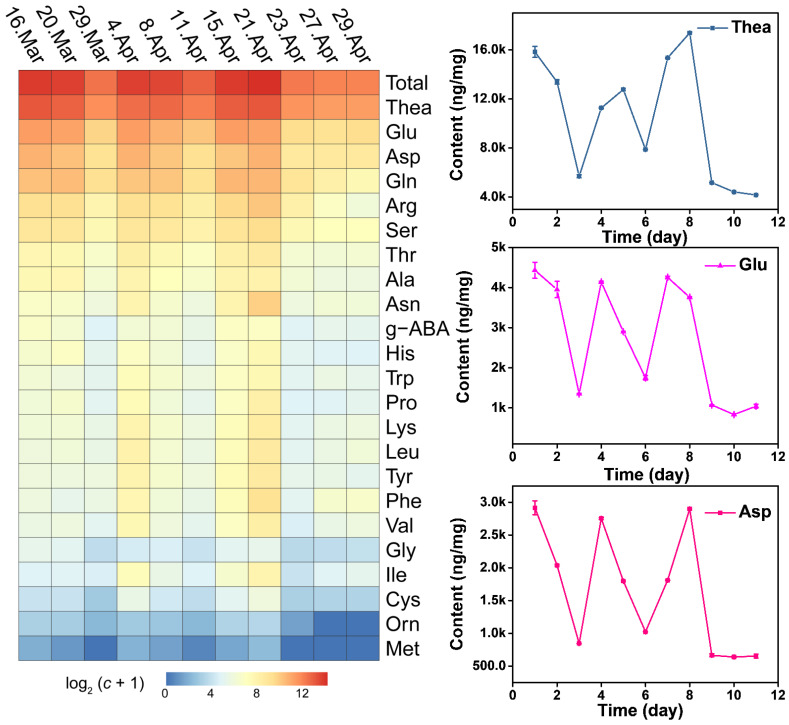
Heatmap of AAs detected from XYMJ green teas and dynamic changes of Thea, Glu, and Asp plucked from March 16, March 20 (Spring Equinox), March 29, April 4 (Pure Brightness), April 8, April 11, April 15, April 21 (Grain Rain), April 23, April 27 to April 29, 2021.

**Figure 5:**
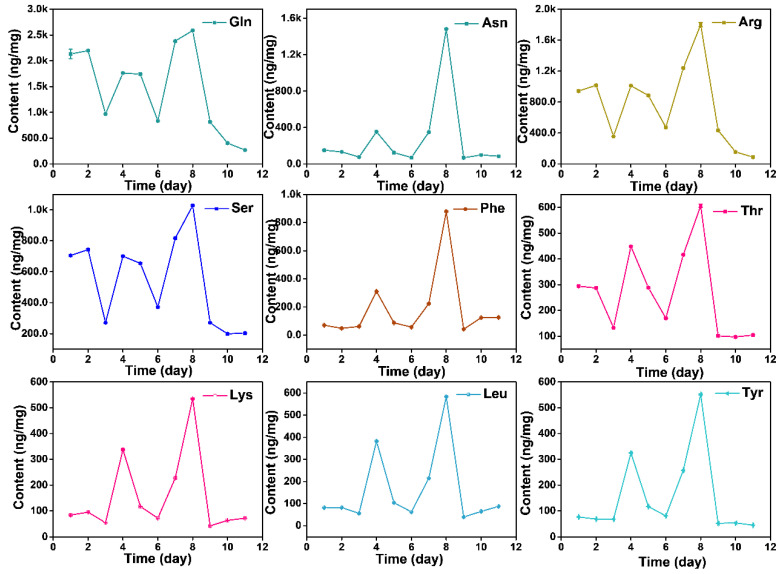
Dynamic changes of AAs (Gln, Asn, Arg, Ser, Phe, Thr, Lys, Leu, and Tyr) from March 16, March 20 (Spring Equinox), March 29, April 4 (Pure Brightness), April 8, April 11, April 15, April 21 (Grain Rain), April 23, April 27 to April 29, 2021.

**Figure 6:**
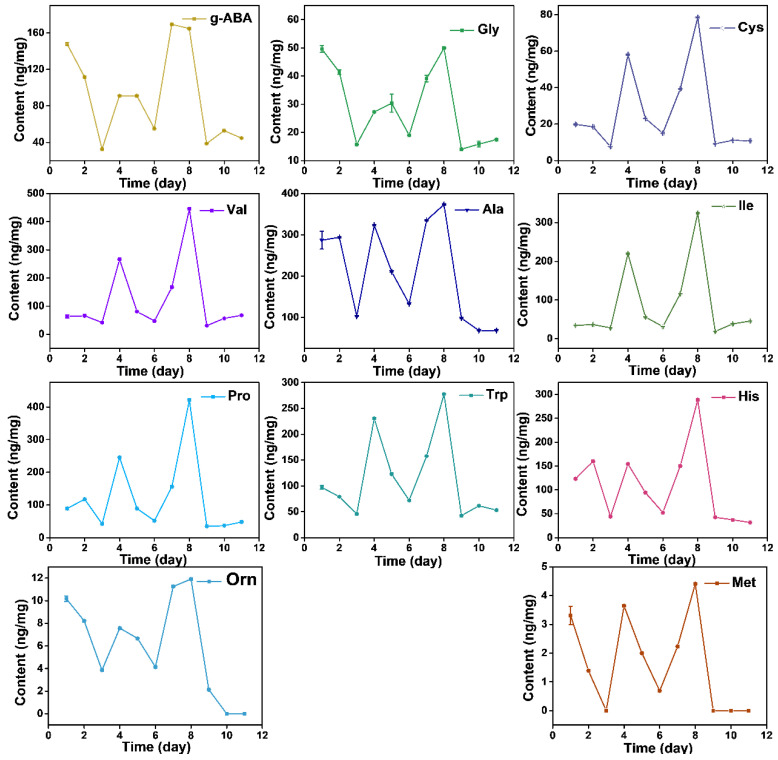
Dynamic changes of AAs (g-ABA, Gly, Cys, Val, Ala, Ile, Pro, Trp, His, Orn, and Met) from March 16, March 20 (Spring Equinox), March 29, April 4 (Pure Brightness), April 8, April 11, April 15, April 21 (Grain Rain), April 23, April 27 to April 29, 2021.

The content of free AAs displays dynamic changes during the growth process, while it did not show a linear increase relationship. Total AAs presented a decrease-increase-decrease-increase-decrease pattern from day before Spring Equinox to nine days after Grain Rain (heatmap in Figure 4). The total free AAs content cumulated to the highest value at Grain Rain and dropped to the lowest after Grain Rain (Table 1 and Table 2). Dynamic changes of total AAs contents from March 16 to April 29 indicates that Pure Brightness and Grain Rain are the key growth process in XYMJ processing, with a positive effect on tea quality and AA amounts. Thea, the most abundant AAs, exhibited the highest content on Grain Rain, and the second peak of Thea content appeared on the day before Spring Equinox (Figure 4). The concentration of Asn, Phe, Thr, Lys, Leu, Ala, Ile, Tyr, Val, Pro, Trp, Cys and Met under different growing periods followed a similar temporal pattern as that of the concentration achieved at the second highest level on Pure Brightness day (Figure 5 and Figure 6). For Asp, Glu, Gln, Ala, g-ABA, Gly, and Orn, a smaller peak appeared on the day before Spring Equinox (Figure 4, Figure 5 and Figure 6). Arg, Ser, and His and displayed a first peak on Spring Equinox (Figure 5 and Figure 6).

### 2.4. Sweet and Umami AAs Contributes to the Quality of XYMJ

Sweet (Pro) and umami amino acids (Asp, Glu, and Thea) were the four major AAs. Gln, Asn, Arg, Ser, Phe, Thr, Tyr, Lys, and Leu were in the second gradient (heatmap in Figure 4). Thus, the high contents of Thea, Glu, Gln, and Asp, which mainly contribute to sweet and umami tastes of green tea, and Ala and Pro exist as auxiliary taste substances, ensures the quality of XYMJ. Consistent with previous studies, Thea was the most abundant AA in green tea, accounting for approximately 50% of total free AAs. In general, free AAs, especially Thea, are responsible for the tea’s umami taste, and the taste intensity increases with the AA concentration. In XYMJ, the results of this study indicated that Thea content was the most abundant, ranging from 5.7 to 17.38 mg/g of the dry weight and accounts for about 44–61% of the total free AAs content in tea leaves from the day before Spring Equinox to Grain Rain (Table 1 and Table 2). Thea exhibited a drastic decrease after Grain Rain (Thea curve in Figure 4), indicating that the long growth time caused consumption of Thea, probably because of the catalysis of endogenous enzymes in the tea conversion process. Moreover, the content of almost all free AAs tended to decrease after Grain Rain. Compared with Pure Brightness, Thea increased significantly in Grain Rain (1.5 times) indicating Grain Rain is a better growth period than Pure Brightness for the purpose of umami tasting XYMJ tea (Thea curve in Figure 4).

Previous research works showed that Gly, Ala, Val, Leu, Ile, and Phe, only have one carboxylic acid group and one amino group with hydrocarbon side chains, have significant antioxidant activity; in addition, AAs with an extra amine group, a thiol group, or a thioether group have better antioxidant activity, Arg, Cys, Lys, Met, and Try, which have obvious electroactivity and undergo oxidation [22]. Although AAs, such as Arg, His, Ile, Leu, Phe, Lys, Tyr, and Val in small amounts (5–14% of total AAs depends on growth stage), will bring about the bitterness and reduce the taste of the comix of XYMJ green tea, the antioxidant activity of the above bitter AAs will be beneficial in maintaining the health benefits for their antioxidant activity.

### 2.5. Stable Weather Conditions Guarantees a Regular Plucking Season

**Figure 7:**
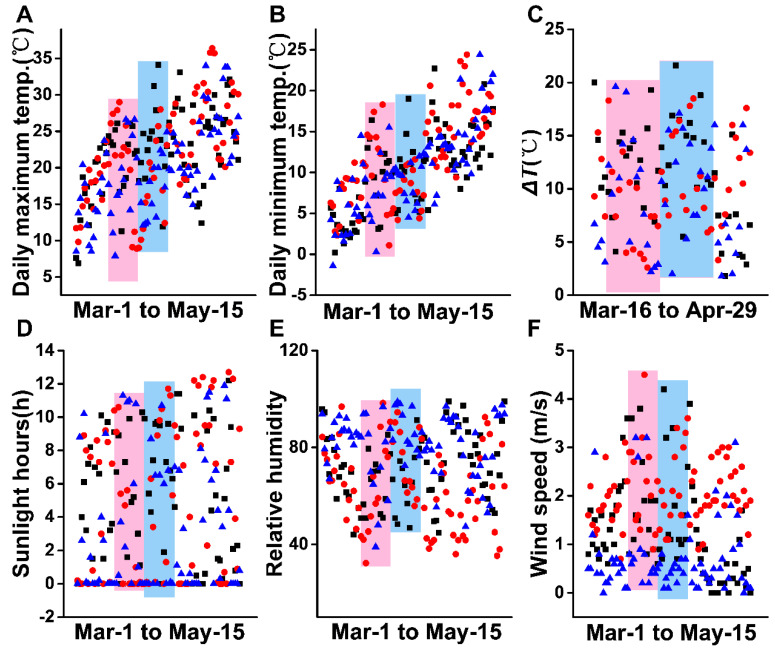
Climate conditions in 2019–2021. Black rectangle points, red dots, and blue triangles represent climatic parameters in 2019, 2020, and 2021, respectively. The red rectangles represent the period from Spring Equinox to Pure Brightness Day, and the blue rectangles represent the period from Pure Brightness Day to Grain Rain. (**A**) Daily maximum temperature; (**B**) daily minimum temperature; (**C**) Δ*T*; (**D**) sunlight hours; (**E**) relative humidity; (**F**) wind speed of Wulidian County of Pingqiao District, in Xinyang County, from March 1 to May 15.

The above results indicate that XYMJ green tea can be plucked from Spring Equinox to Grain Rain, especially on Pure Brightness and Grain Rain Day in 2021. It is reported that the seasonal differences of teas having an effect on their metabolites, teas with different harvest years but the same season, were also an important factor that affects the quality of teas [23]. Especially, average climatic temperature, altitude, and tea leaves picking time significantly influence the quality of tea [24]. By records and statistics of the climate recipes in three recent years, a condition was identified in which the daily environmental temperature gradient varies in a large range from 0 to 35 °C, but the overall trend is capable of a constant model in years 2019–2021 from March 1 to May 15 (Figure 7A,B). Figure 7C shows the magnitudes of temperature change (Δ*T*) within each day through March 16 to April 29. For this growth period, Δ*T* likely exceeded 20 °C, while the changes over the past three years tend to be uniform. Similarly, sunlight hours and the relative humidity shared the same tendency for three years (Figure 7D,E). The wind speeds in 2021 were generally lower than those for 2020 and 2019, but have a slight influence on the quality of XYMJ green tea. Therefore, the stable climate conditions from Spring Equinox to Grain Rain ensure the consistency of the harvest season.

## 3. Discussion

Green tea, the most widely consumed tea in China, can benefit human health because of its chemical composition, which includes catechins, theanine, and caffeine [25,26,27]. XYMJ green tea is produced in the middle of China through spreading, green removing, rolling, and drying with the fresh tea leaves as the raw material [28]. The chemical composition, especially for AAs, depends on numerous factors, such as planting area, tea plantation management, processing practices, tea variety, and climate, play the most important, direct role in the characterization and differentiation of tea quality and hence their market prices. Changes in free AA profiles during their growth process contribute to tea’s flavor through certain complex reactions. Dynamic changes in AAs contents in different growth processes involve both increasing contents attributed to protein break-down, hydrolysis, or cleavage and decreasing content due to their consumption as precursors of other compounds. Elucidating AAs changes during the stage of growth will be helpful to promote the quality and commercial value of green tea. Even though extensive researches about AAs contents or dynamic changes have been reported and achieved some improvements in determining the relationship between green tea quality formation and the impact of factors during the stage of green tea growth, some challenges, such as dynamic changes during the growth stage and other types of compounds in organic phases obtained from XYMJ green tea, remain. Therefore, to evaluate tea quality, effective identification of growth stages is crucial.

Our research shows that climate parameters, such as the daily maximum temperature, daily minimum temperature, Δ*T*, sunlight hours, and relative humidity, in the same place kept in a steady condition, ensure the consistent plucking seasons every year. The separation degree of 23 AAs detected by a Hitachi AA analyzer indicates that our experimental method is feasible. Heatmaps were applied to obtain a preliminary overview of the difference contents of 23 AAs. It shows that the total amino acid content is extremely low far from the growth period between Spring Equinox and Grain Rain. Our study shows that growth period has a great influence on the amounts of AAs in XYMJ green tea. Pure Brightness and Grain Rain are the best picking days for XYMJ. The quality will be greatly compromised after Grain Rain. Despite Grain Rain being a better growth period than Pure Brightness for the purpose of umami tasting XYMJ tea, it is not recommended to start plucking tea leaves at Grain Rain. Because 23 AAs decreased significantly after Grain Rain, it is obviously unwise for the tea economy.

Phytosterols are another major component in vegetative oils and the total content of sterols was reported as 1099–2298 mg/kg, 1494–5113 mg/kg, and 1100–1800 mg/kg in oleifera oil, camellia oil and Thea oil, respectively [18]. In canola, phytosterols constitute about 0.5% of the seed oil [29]. It is generally assumed that phytosterols play a key role in stabilizing the cell membranes in plants (as cholesterol does in animals) [30]; however, the chemical composition of sterols from XYMJ tea tree leaves remains elusive. Spinasterol and 22,23-dihydrospinasterol are triterpenoids that are produced by a number of plants [31]. An extract from the leaves of *Mukia maderaspatana* indicated that spinasterol and 22,23-dihydrospinasterol have potential antioxidant properties. A pharmacological study of *Bougainvillea spectabilis* stems reported that spinasterol and 22,23-dihydrospinasterol can be used in herbal medicines against cancer and hepatitis. In this study, we identified spinasterol and 22,23-dihydrospinasterol from XYMJ green tea leaves, which showed larger amounts than the other compounds in the ethanol extract. It suggests that spinasterol and 22,23-dihydrospinasterol from green tea tree leaves are commonly existent.

## 4. Materials and Methods

### 4.1. Chemicals and Reagents

AA stock solutions of 200 mg/L were prepared in 0.1 M HCl and stored at 4 °C. Diluted solutions were prepared weekly. OPA and mercaptoethanol were purchased from Sigma-Aldrich. Acetonitrile and methanol, HPLC grade, were purchased from Romil (Cambridge, UK). Hydrochloric acid, sodium hydroxide, boric acid, and disodium phosphate were obtained from Hitachi (Tokyo, Japan). A 40 mM phosphate buffer (pH 7.5), prepared from disodium phosphate and hydrochloric acid, and a 0.4 M boric acid/borate buffer (pH 9.5), were prepared. The derivatization reagent was freshly prepared everyday by dissolving 50 mg of OPA in 1.5 mL of mercaptoethanol. Ultrapure water was used throughout and was obtained from a Milli-Q system from Millipore (Milford, MA, USA).

### 4.2. Tea Samples

In this study, one bud with one leaf of XYMJ green teas were plucked from Wulidian County of Pingqiao District, in Xinyang County, Henan Province, China (N32°9′9″/114°17′58″ E) on March 16, March 20 (Spring Equinox), March 29, April 4 (Pure Brightness), April 8, April 11, April 15, April 21 (Grain Rain), April 23, April 27, and April 29, 2021, respectively. All XYMJ green teas plucked from the above days were then manufactured according to traditional processing steps [32], including fixing, rolling, shaping, and drying. Fresh leaves were fixed in a hot pan (180 °C) with a rolling bamboo brush for 4 min. After cooling to room temperature, the fixated leaves were rolled in a rolling machine for 20 min, and then, the rolled leaves were shaped with a shaping machine at 60 °C for 20 min. Finally, the shaped leaves were dried using hot air at 90 °C until fully dried. Then, all of the samples were harvested and were immediately freeze-dried under vacuum, and stored at −40 °C for further analysis.

### 4.3. Determination of Free AAs

The Chinese National Standard GB/T 30987-2020 method was used for the analysis of free AAs with slight modifications [33]: Each individual tea sample in Section 4.2 (100 mg) was ground into a homogenous powder and extracted with 4 mL of distilled water at 80 °C for 25 min. After it was cooled at room temperature, the infusion was collected by centrifuging the solid matter at 13,201*× g* for 15 min, returning the volume up to 5 mL again with distilled water. Following this, 1 mL of the obtained filtrate (as the infused tea sample) was diluted with water to 10 mL and used for precolumn reaction with 5 mL derivatization reagents and proceeded to measurement. The ultraviolet–visible spectroscopy (UV–Vis) detection wavelengths were set at 570 and 440 nm. The injection volume was 50 µL. The timeline for each individual step of the process preparation from tea powder infusion to the AA analyzer injection was set to 90 min as the total experiment time. The other parameters were shown in Supplementary Materials.

### 4.4. Extraction and Isolation of Spinasterol and 22,23-Dihydrospinasterol

The air-dried and powdered XYMJ green teas (3.5 kg) were extracted with EtOH (3 × 25 L, 7 days each) at room temperature [34]. Evaporation of the solvent gave a residue (120 g) that was subjected to silica gel column chromatography (petroleum ether/ethyl acetate, 100:1, 80:1, 50:1, 20:1, 10:1, 5:1, 2:1, and 0:1, *v*/*v*) to afford eight crude fractions (Fr. 1−8) based on TLC analysis. Fr. 4 (2.8 g) was purified by silica gel column chromatography employing petroleum ether/ethyl acetate (25:1–10:1) to afford white solids which after recrystallization from petroleum ether/ethyl acetate.

### 4.5. AA Analyzer Analysis

AAs were determined using a Hitachi L-8900 AA Analyzer (Tokyo, Japan) [3]. Hitachi separation columns utilize 4.6 mm I.D. × 60 mm L packed with Hitachi custom ion exchange resin (particle size: 3 µm). All buffers, reagents, and routine maintenance parts are accessible from the front panel, and EZChrom Elite software provides full instrument control and data processing. The other parameter settings are available in the Supplementary Materials.

### 4.6. ^1^H-NMR (600 MHz, CDCl_3_) and ^13^C-NMR (151 MHz, CDCl_3_) of Spinasterol and 22,23-Dihydrospinasterol

Spinasterol ^1^H-NMR (600 MHz, CDCl_3_) *δ* 5.16 (overlap, 1H H-7), 5.16 (overlap, 1H, H-22), 5.04 (dd, *J* = 15.1, 8.8 Hz, 1H, H-23), 3.60 (m, 1H, H-3) allylic protons, 1.03 (d, *J* = 7.0 Hz, 3H, -CH_3_), 0.85 (t, *J* = 6.8 Hz, 3H, -CH_3_), 0.82 (d, *J* = 6.9 Hz, 3H, -CH_3_), 0.81 (d, *J* = 6.9 Hz, 3H, -CH_3_), 0.80 (s, 3H, -CH_3_), 0.54 (s, 3H, -CH_3_). ^13^C-NMR (151 MHz, CDCl_3_) *δ* 37.15, 31.44, 71.03, 37.95, 40.25, 29.65, 117.40, 139.54, 49.27, 34.2, 21.55, 39.47, 45.82, 55.12, 23.03, 28.53, 55.89, 11.99, 13.06, 40.8, 21.12, 138.18, 129.42, 51.28, 31.89, 18.92, 21.40, 25.42, 12.27.

22,23-dihydrospinasterol ^1^H-NMR (600 MHz, CDCl_3_) *δ* 5.16 (m, 1H, H-7), 3.60 (m, 1H, H-3) allylic protons, 0.93 (d, *J* = 7.0 Hz, 3H, -CH_3_), 0.85 (t, *J* = 6.8 Hz, 3H, -CH_3_), 0.82 (d, *J* = 6.9 Hz, 3H, -CH_3_), 0.81 (d, *J* = 6.9 Hz, 3H, -CH_3_), 0.80 (s, 3H, -CH_3_), 0.55 (s, 3H, -CH_3_). ^13^C-NMR (151 MHz, CDCl_3_) *δ* 37.15, 31.44, 71.03, 37.95, 40.25, 29.65, 117.42, 139.60, 49.44, 34.2, 21.12, 39.58, 43.38, 55.89, 22.98, 27.98, 55.89, 11.86, 13.06, 36.60, 18.92, 33.89, 26.17, 45.82, 29.14, 19.85, 19.05, 22.98, 11.99.

### 4.7. Statistical Analysis

Two-factor experiments with randomized design were applied to this study. All data were presented as the mean ± standard deviation (SD) of triplicate experiments. The peaks presented in the analyte were evaluated by their retention times compared to the control sample. The heatmap was drawn using R software (pheatmap package). The date of the heatmap was normalized with log_2_(*C*+1), where *C* represents the concentration of amino acid, unit: ng/mg.

## 5. Conclusions

In conclusion, the harvest seasons of XYMJ green tea have great influence on the contents of AAs and spinasterol and 22,23-dihydrospinasterol are commonly existent. The dynamic changes of 23 AAs could bias tea quality, leading to the superior economics for XYMJ green tea market. These results provide important insights for the development of a general method of testing 23 AAs to determine the plucking seasons. They stress the key role of Pure Brightness and Grain Rain during the tea growing season and explain the reason for tea quality decline after Grain Rain.

## Figures and Tables

**Table 1:** Average contents of 23 AAs from March 16, March 20 (Spring Equinox), March 29, April 4 (Pure Brightness), April 8 to April 11. Units: ng/mg.

	Ret. Times	AA	3.16 (ng/mg)	3.20	3.29	4.4	4.8	4.11
1	9.753	Asp	2916.83	2037.42	843.2	2752.88	1796.81	1022.79
2	14.38	Thr	294.2	287.09	133.01	448.29	288.66	170.06
3	15.833	Ser	705.32	743.31	270.84	700.89	654.58	371.82
4	19.66	Glu	4432.11	3950.58	1349.34	4139.97	2895.17	1752.67
5	33.86	Gly	49.62	41.41	15.73	27.23	30.4	19.01
6	35.78	Ala	286.81	294.04	102.64	323.2	210.83	132.95
7	41.74	Val	63.08	65.89	41.85	266.1	80.71	47.75
8	43.147	Cys	19.88	18.51	7.64	58.09	23.12	15.04
9	44.567	Met	3.31	1.39	0	3.64	2	0.69
10	47.94	Ile	34.56	36.6	28.02	219.75	56.09	31.41
11	49.613	Leu	81.43	81.48	55.63	381.8	103.77	62.19
12	51.267	Tyr	76.42	68.3	67.78	324.59	116.86	80.54
13	53.887	Phe	70.16	48.14	61.24	310.73	87.26	56.75
14	61.24	g-ABA	147.73	111.46	32.35	91	91.01	54.93
15	73.213	Orn	10.17	8.22	3.85	7.57	6.66	4.13
16	74.9	Lys	83.77	94.92	54.47	337.72	117.3	71.58
17	77.747	His	123.28	159.85	43.86	154.24	94.36	52.27
18	87.16	Arg	943.04	1016.36	357.46	1010.89	885.59	473.69
19	31.747	Pro	89.14	117.44	41.77	245.67	88.82	51.49
20	18.253	Asn	151.06	132.87	76.5	353.18	124.3	71.72
21	21.18	Gln	2133	2196.37	971.44	1762.92	1740.42	833.52
22	63.5	Trp	97.03	78.96	45.93	230.54	122.72	71.96
23	24.853	Thea	15,827.92	13,375.62	5689.61	11,253.39	12,762.7	7864.51

**Table 2:** Average contents of 23 AAs from April 15, April 21 (Grain Rain), April 23, April 27 to April 29, 2021. Units: ng/mg.

	Ret. Times	AA	4.15	4.21	4.23	4.27	4.29
1	9.753	Asp	1810.07	2897.93	663.7	637.78	652.41
2	14.38	Thr	415.84	606.1	101.47	97.17	104.76
3	15.833	Ser	816.86	1027.64	270.48	199.09	203.31
4	19.66	Glu	4254.7	3755.79	1073.08	833.69	1042.77
5	33.86	Gly	39.08	49.94	14.04	15.85	17.43
6	35.78	Ala	335.01	373.34	98.09	68.31	68.35
7	41.74	Val	167.09	444.92	30.78	56.77	67.67
8	43.147	Cys	39.33	78.53	9.22	11.1	10.86
9	44.567	Met	2.23	4.41	0	0	0
10	47.94	Ile	116	324.18	18.88	38.33	45.76
11	49.613	Leu	214.68	583.55	38.84	64.29	87.02
12	51.267	Tyr	257.16	551.5	51.74	53.5	44.67
13	53.887	Phe	224.08	878.66	41.94	123.39	126.14
14	61.24	g-ABA	169.36	164.79	38.46	52.68	44.48
15	73.213	Orn	11.25	11.91	2.13	0	0
16	74.9	Lys	227.37	535.32	41.79	63.4	71.48
17	77.747	His	150.28	288.79	42.43	37.16	31.51
18	87.16	Arg	1238.09	1799.38	435.14	155.37	88.76
19	31.747	Pro	156.03	421.33	34.38	36.36	47.67
20	18.253	Asn	349.9	1482.61	68.1	99.46	84.87
21	21.18	Gln	2382.53	2587.07	813.89	403.73	269.42
22	63.5	Trp	157.66	277.55	42.47	61.49	53.08
23	24.853	Thea	15,336.19	17,382.16	5167.52	4413.95	4168

## Data Availability

Not applicable.

## Supplementary Materials

The following are available online at https://www.mdpi.com/article/10.3390/molecules27113562/s1, Table S1: Composition of Buffer Solution for Physiological Fluid Assay.

## Sample Availability

Samples of the compounds are not available from the authors.
