# Supplementary material for: Dynamic Variation of Amino Acid Contents and Identification of Sterols in Xinyang Mao Jian Green Tea
Source: Molecules. 2022 Jun 1;27(11):3562. doi: 10.3390/molecules27113562 (PMC9182030; doi:10.3390/molecules27113562)
Supplement: Supplementary file 1 [file molecules-27-03562-s001.zip › molecules-1734133-supplementary.pdf]

Dynamic Variation of Amino Acid Contents and Identification of Sterols in Xinyang Mao Jian Green Tea

Meng Sun [1], Fangfang Yang [2], Wanying Hou [1], Shuangfeng Jiang [1,3], Runqi Yang [1], Wei Zhang [1], Mingjie Chen [1], Yuhang Yan [1], Yuxin Tian [1] and Hongyu Yuan [1,*]

Henan Key Laboratory of Tea Plant Biology, College of Life Sciences, Xinyang Normal University, Xinyang 464000, China; sunmeng2010abc@163.com (M.S.); battc9839bgrg0@163.com (W.H.); jshuangfeng@163.com (S.J.); yangrunqi246875@163.com (R.Y.); chawenhua2009@163.com (W.Z.); mjchen006@163.com (M.C.); 17839865339@163.com (Y.Y.); 15516796003@163.com (Y.T.)

[2] Guangxi-ASEAN Food Inspection and Testing Center, Nanning 530029, China; yangfangfang18@126.com

[3] Xinyang Academy of Agricultural Sciences, Xinyang 464000, China

* Correspondence: yhyuan@xynu.edu.cn; Tel.: +0376-6391289

Instruments：

Hitachi L-8900 Amino Acid Analyzer (Tokyo, Japan)

Standard solutions：

Wako Amino Acids Mixture Standard Solution, Type B 012-08643

Wako Amino Acids Mixture Standard Solution, Type AN-2 015-14461

Preparing standard solution for analysis：

Taking 0.100 mL of the above amino acid standard solutions and mixing totally, then adding water in constant volume to 1.000 mL.

Chromatographic column:

4.6 mm I.D.×60 mm L packed with Hitachi custom ion exchange resin (Partical size: 3 μm)； Hitachi l-8900 amino acid analyzer separation column: 4.6 mm × 60 mm, cation exchange resin (particle size: 3 μm); Net analysis time: approximate 90 min, 110 min/ cycle.

Flow rate:

Separation buffer: 0.35 mL/min, reaction solution: 0.3 mL/min.

Detection wavelength：570 nm, 440 nm

Analysis method: Standard analysis method of L-8900 amino acid analyzer system

Separation buffer:

Table S1. Composition of Buffer Solution for Physiological Fluid Assay

Name	PF-1	PF-2	PF-3	PF-4	PF-RG
Vessel(buffer)	B1	B2	B3	B4	B6
Lithium concentration (N)	0.09	0.255	0.721	1.00	0.20
1. Distilled water (approx.)	700 mL	700 mL	700 mL	700 mL	700 mL
2. Lithium citrate ($4H_2O$)	5.73 g	9.80 g	8.79 g	9.80 g	-
3. Lithium chloride	1.24 g	6.36 g	26.62 g	38.15 g	-
4. Citric acid (H_2O)	19.90 g	12.00 g	11.27 g	3.30 g	-
5. Lithium hydroxide	-	-	-	-	-
6. Ethanol	30.0 mL	30.0 mL	100.0 mL	-	30.0 mL
7. Thiodiglycol	5.0 mL	5.0 mL	-	-	-
8. Benzyl alcohol	-	-	3.0 mL	-	-
9. Brij-35*	4.0 mL	4.0 mL	4.0 mL	4.0 mL	4.0 mL
10. pH (nominal)	2.8	3.7	3.6	4.1	-
11. Total (adjust)	1.0 L	1.0 L	1.0 L	1.0 L	1.0 L
12. Caprylic acid	0.1 mL	0.1 mL	0.1 mL	0.1 mL	0.1 mL
*Dissolve 25 g into 100mL of distilled water.					

Reaction solution:

Vessel	Step	Reagent	Measurement
R1(ninhydrin)	1	Propylene glycol monomethyl ether	979 mL
	2	Ninhydrin	39 g
	3	Nitrogen bubbling, dissolution	5 min minimum
	4	Sodium borohydride	81 mg
	5	Nitrogen bubbling	30 min minimum
		Density	0.96
R2 for ninhydrin buffer solution	1	Distilled water	336 mL
	2	Lithium acetate dihydrate	204 g
	3	Glacial acetic acid	123 mL
	4	Propylene glycol monomethyl ether	401 mL
	5	Total	1000 mL
	6	Nitrogen bubbling	10 min minimum
		Density	0.96
R3 for 5% of Ethanol	1	Distilled water	900 mL
	2	Ethanol	50 mL
	3	Total	1000 mL
		Density	1.00

Elution gradient:

Analysis Condition

B1:PF-1
B2:PF-2
B3:PF-3
B4:PF-4
B5:H_2O

R1:Nin
R2:Nin-Buffer
R3:5%Ethanol

Time (min)	%B1	%B2	%B3	%B4	%B5	%B6	Pump 1 Flow rate (mL/min)	Column Temp.	%R1	%R2	%R3	Pump 2 Flow rate (mL/min)
0.0	100	0	0	0	0	0	0.350	38	50	50	0	0.300
2.0								30				
21.5	100	0	0	0	0	0						
21.6	80	20	0	0	0	0		60				
33.5	70	30	0	0	0	0						
33.6	10	90	0	0	0	0						
36.5								40				
43.5	10	90	0	0	0	0						
43.6	0	100	0	0	0	0						
50.5	0	100	0	0	0	0		70				
50.6	0	0	100	0	0	0						
68.4								45				
69.5	0	0	100	0	0	0						
69.6	60	0	0	40	0	0						
75.0	60	0	0	40	0	0						
75.1	0	0	0	100	0	0						
82.0	0	0	0	100	0	0						
82.1	0	20	0	80	0	0						
92.5								70				
99.5	0	20	0	80	0	0						
99.6	0	0	0	100	0	0						
112.5	0	0	0	100	0	0						
112.6	0	0	0	0	0	100						
116.0									50	50	0	
116.1									0	0	100	
121.5	0	0	0	0	0	100						
121.6	100	0	0	0	0	0						
125.0								38				
126.0									0	0	100	
126.1									50	50	0	
148.0	100	0	0	0	0	0						
