# Supplementary material for: Dynamic Variation of Amino Acid Contents and Identification of Sterols in Xinyang Mao Jian Green Tea
Source: Molecules. 2022 Jun 1;27(11):3562. doi: 10.3390/molecules27113562 (PMC9182030; doi:10.3390/molecules27113562)
Supplement: Supplementary file 1 [file molecules-27-03562-s001.zip › pf-sm-chaye-yp202129670-sample-1-6-6ul-2021-7-3 11-34-53.pdf]

Hitachi Amino Acid Analyzer Report

Data File: E:\data\20210701 PF\PF-sm-chaye-yp202129670-sample-1-6-6ul-2021-7-3 11-34-53.dat
Method: E:\data\20210701 PF\sm-chaye.met
Sample ID: {Sample ID}
Vial Number: 163 Inj. Volume(uL): 6

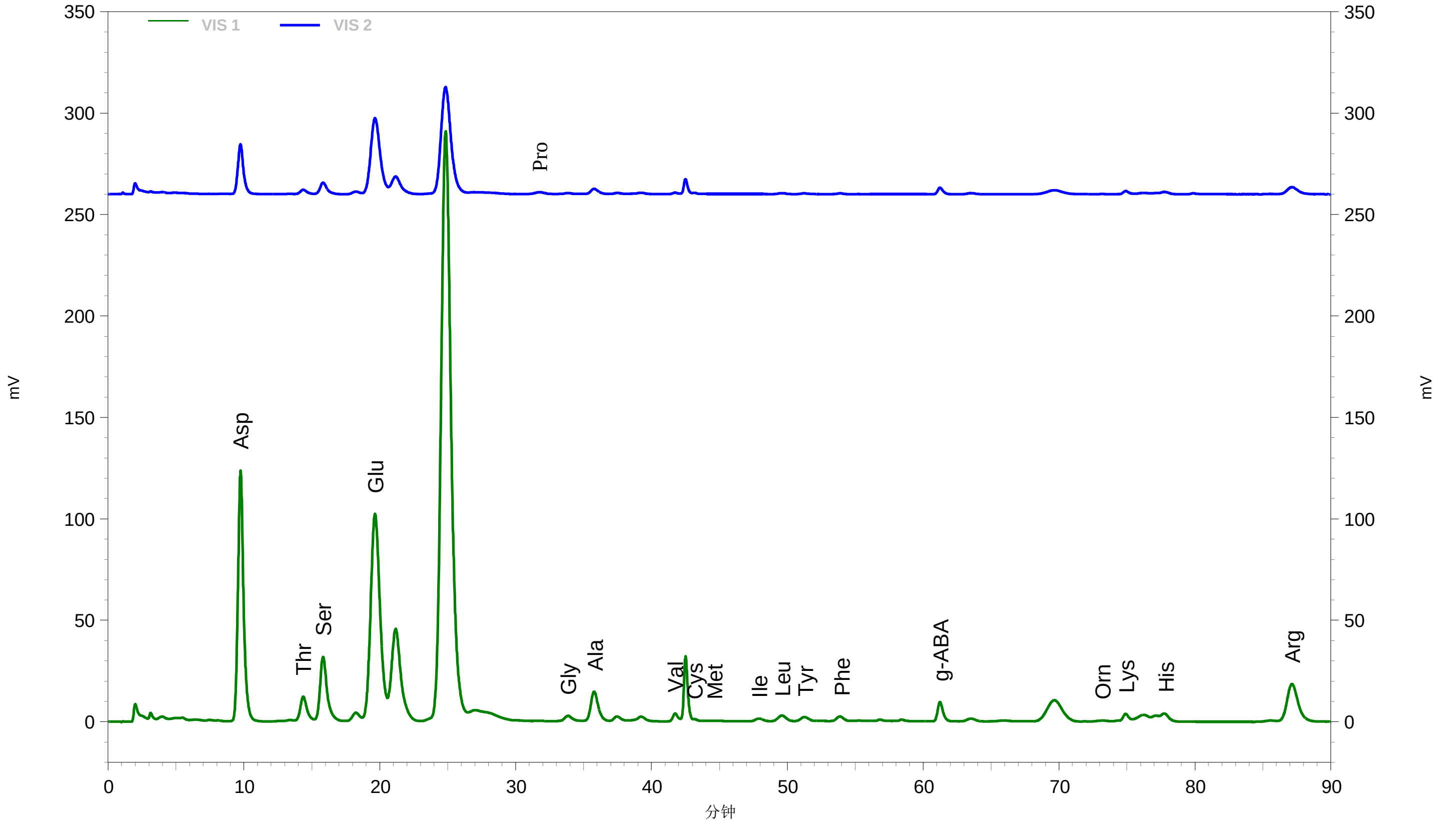

VIS 1 result

Peak #	Retention Time	Name	Area	Resolution	Mol concentration (nmol/mg)	CalcMol (ng/mg)
10	9.753	Asp	14144134	2.24	16.09	2142.23
12	14.380	Thr	1638000	1.11	1.78	212.48
13	15.833	Ser	4573329	1.63	4.96	520.90
15	19.660	Glu	19918036	1.20	22.10	3250.80
19	33.860	Gly	390642	2.71	0.41	31.04
20	35.780	Ala	2167361	1.92	2.26	201.03
23	41.740	Val	392261	2.76	0.39	46.10
25	43.147	Cys	70295	0.51	0.06	14.84
26	44.567	Met	17604	0.94	0.02	2.56
27	47.940	Ile	200009	3.33	0.19	25.45
28	49.613	Leu	461492	1.56	0.46	59.85
29	51.267	Tyr	317616	1.48	0.32	57.38
30	53.887	Phe	313544	2.55	0.32	52.66
35	61.240	g-ABA	1043374	4.05	1.08	111.73
39	73.213	Orn	74473	2.12	0.05	6.95
40	74.900	Lys	489984	1.64	0.42	62.06
43	77.747	His	685259	0.29	0.63	97.26
45	87.160	Arg	4233875	1.04	4.22	735.92
Sum			51131288		55.77	

VIS 2 result

Peak #	Retention Time	Name	Area	Resolution	Mol concentration (nmol/mg)	CalcMol (ng/mg)
8	31.747	Pro	143038	5.40	0.52	59.84
Sum			143038		0.52	
