# Supplementary material for: Dynamic Variation of Amino Acid Contents and Identification of Sterols in Xinyang Mao Jian Green Tea
Source: Molecules. 2022 Jun 1;27(11):3562. doi: 10.3390/molecules27113562 (PMC9182030; doi:10.3390/molecules27113562)
Supplement: Supplementary file 1 [file molecules-27-03562-s001.zip › pf-sm-chaye-yp202129670-sample-1-6-6ul-2021-7-3 11-34-53_REPORT-asn.pdf]

Hitachi Amino Acid Analyzer Report

Data File: E:\data\20210701 PF\PF-sm-chaye-yp202129670-sample-1-6-6ul-2021-7-3 11-34-53.dat
Method: E:\data\20210701 PF\sm-asn.met
Sample ID: {Sample ID}
Vial Number: 163 Inj. Volume(uL): 6

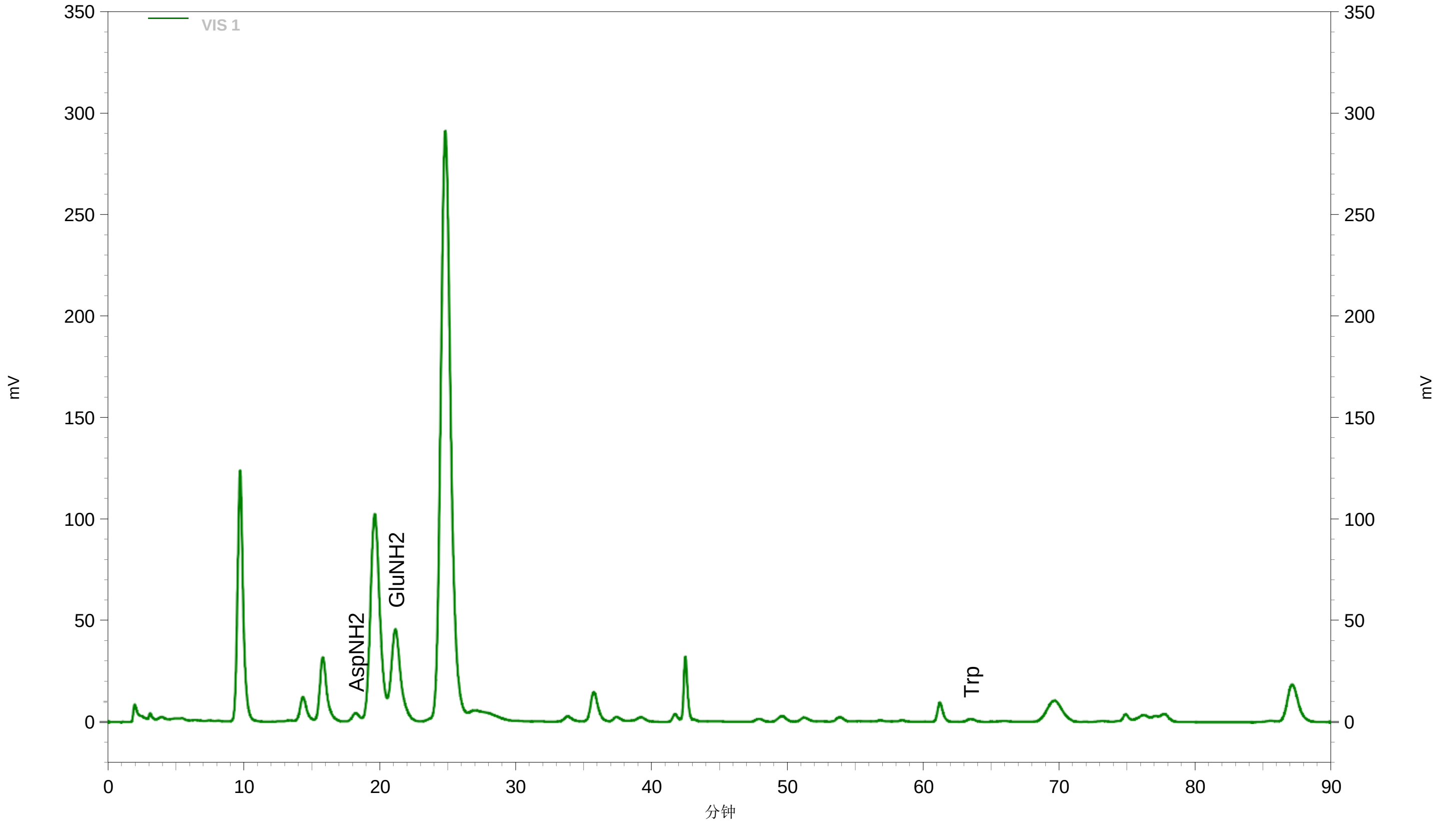

VIS 1 result

Peak #	Retention Time	Name	Area	Resolution	Mol concentration (nmol/mg)	CalcMol (ng/mg)
14	18.253	AspNH2	594463	2.48	0.83	109.73
16	21.180	GluNH2	9001338	1.18	11.00	1607.15
36	63.500	Trp	227287	2.40	0.36	72.57
Sum			9823088		12.19	

VIS 2 result

Peak #	Retention Time	Name	Area	Resolution	Mol concentration (nmol/mg)	CalcMol (ng/mg)
