# Supplementary material for: Dynamic Variation of Amino Acid Contents and Identification of Sterols in Xinyang Mao Jian Green Tea
Source: Molecules. 2022 Jun 1;27(11):3562. doi: 10.3390/molecules27113562 (PMC9182030; doi:10.3390/molecules27113562)
Supplement: Supplementary file 1 [file molecules-27-03562-s001.zip › pf-sm-chaye-yp202129670-sample-1-7-6ul-2021-7-2 7-31-03_REPORT.pdf]

Hitachi Amino Acid Analyzer Report

Data File: E:\data\20210701 PF\PF-sm-chaye-yp202129670-sample-1-7-6ul-2021-7-2 7-31-03.dat
Method: E:\data\20210701 PF\sm-chaye.met
Sample ID: {Sample ID}
Vial Number: 151 Inj. Volume(uL): 6

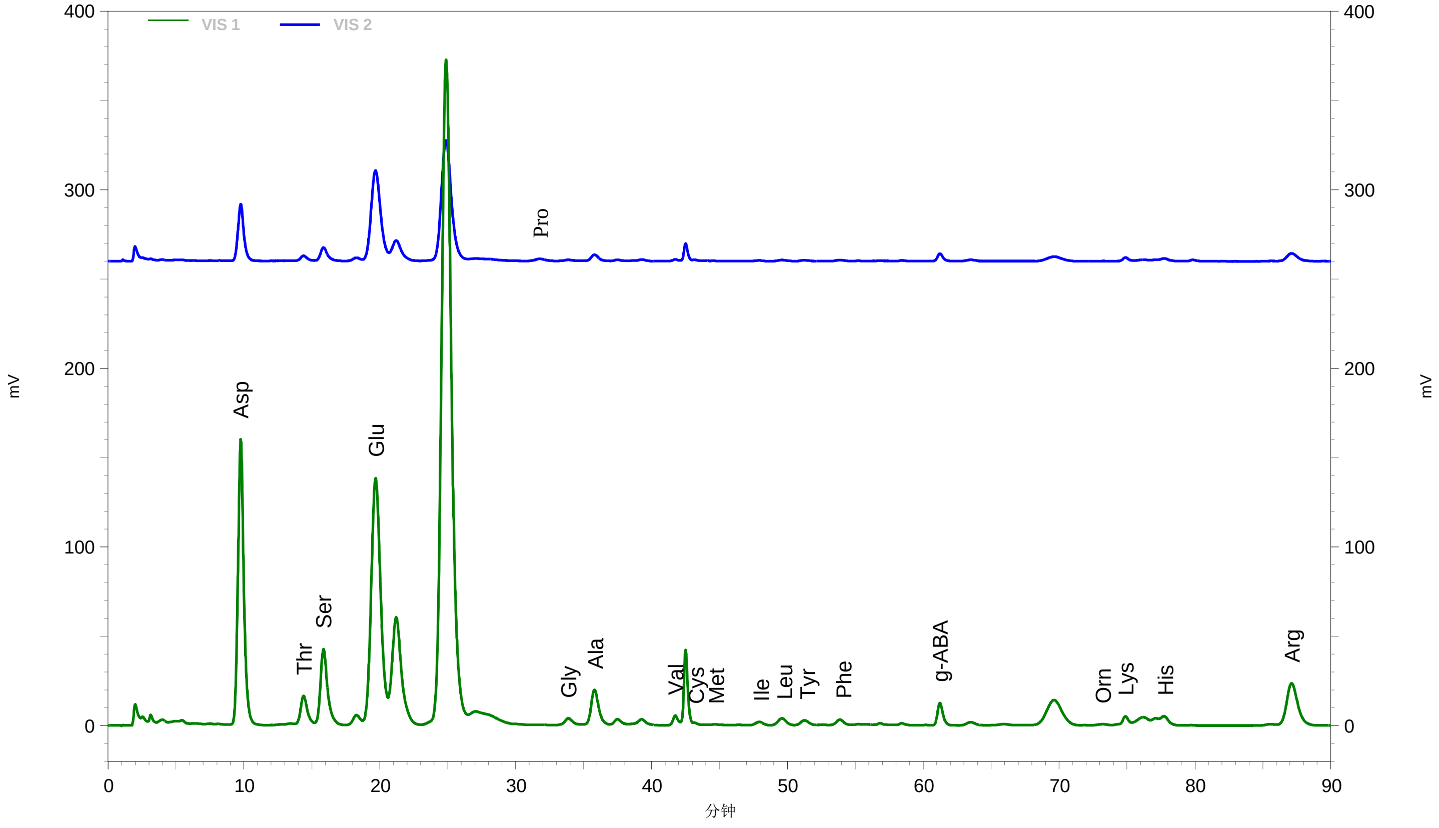

VIS 1 result

Peak #	Retention Time	Name	Area	Resolution	Mol concentration (nmol/mg)	CalcMol (ng/mg)
11	9.767	Asp	19258446	1.54	21.91	2916.83
14	14.400	Thr	2267927	0.91	2.47	294.20
15	15.867	Ser	6192509	1.61	6.71	705.32
17	19.693	Glu	27156121	1.17	30.13	4432.11
21	33.887	Gly	624519	2.62	0.66	49.62
22	35.807	Ala	3092234	1.86	3.22	286.81
25	41.753	Val	536786	2.70	0.54	63.08
27	43.147	Cys	94180	0.67	0.08	19.88
28	44.673	Met	22816	1.32	0.02	3.31
30	47.940	Ile	271574	1.43	0.26	34.56
31	49.613	Leu	627814	1.56	0.62	81.43
32	51.267	Tyr	423064	1.48	0.42	76.42
33	53.873	Phe	417729	2.53	0.42	70.16
39	61.227	g-ABA	1379576	1.37	1.43	147.73
43	73.240	Orn	108954	2.13	0.08	10.17
44	74.887	Lys	661378	1.58	0.57	83.77
47	77.727	His	868616	0.28	0.79	123.28
49	87.120	Arg	5425489	0.96	5.41	943.04
Sum			69429732		75.77	

VIS 2 result

Peak #	Retention Time	Name	Area	Resolution	Mol concentration (nmol/mg)	CalcMol (ng/mg)
8	31.813	Pro	213084	5.27	0.77	89.14
Sum			213084		0.77	
