# Supplementary material for: Dynamic Variation of Amino Acid Contents and Identification of Sterols in Xinyang Mao Jian Green Tea
Source: Molecules. 2022 Jun 1;27(11):3562. doi: 10.3390/molecules27113562 (PMC9182030; doi:10.3390/molecules27113562)
Supplement: Supplementary file 1 [file molecules-27-03562-s001.zip › pf-sm-chaye-yp202129670-sample-10-6ul-2021-7-3 5-58-11_REPORT-.pdf]

Hitachi Amino Acid Analyzer Report

Data File: E:\data\20210701 PF\PF-sm-chaye-yp202129670-sample-10-6ul-2021-7-3 5-58-11.dat
Method: E:\data\20210701 PF\sm-chaye.met
Sample ID: {Sample ID}
Vial Number: 160 Inj. Volume(uL): 6

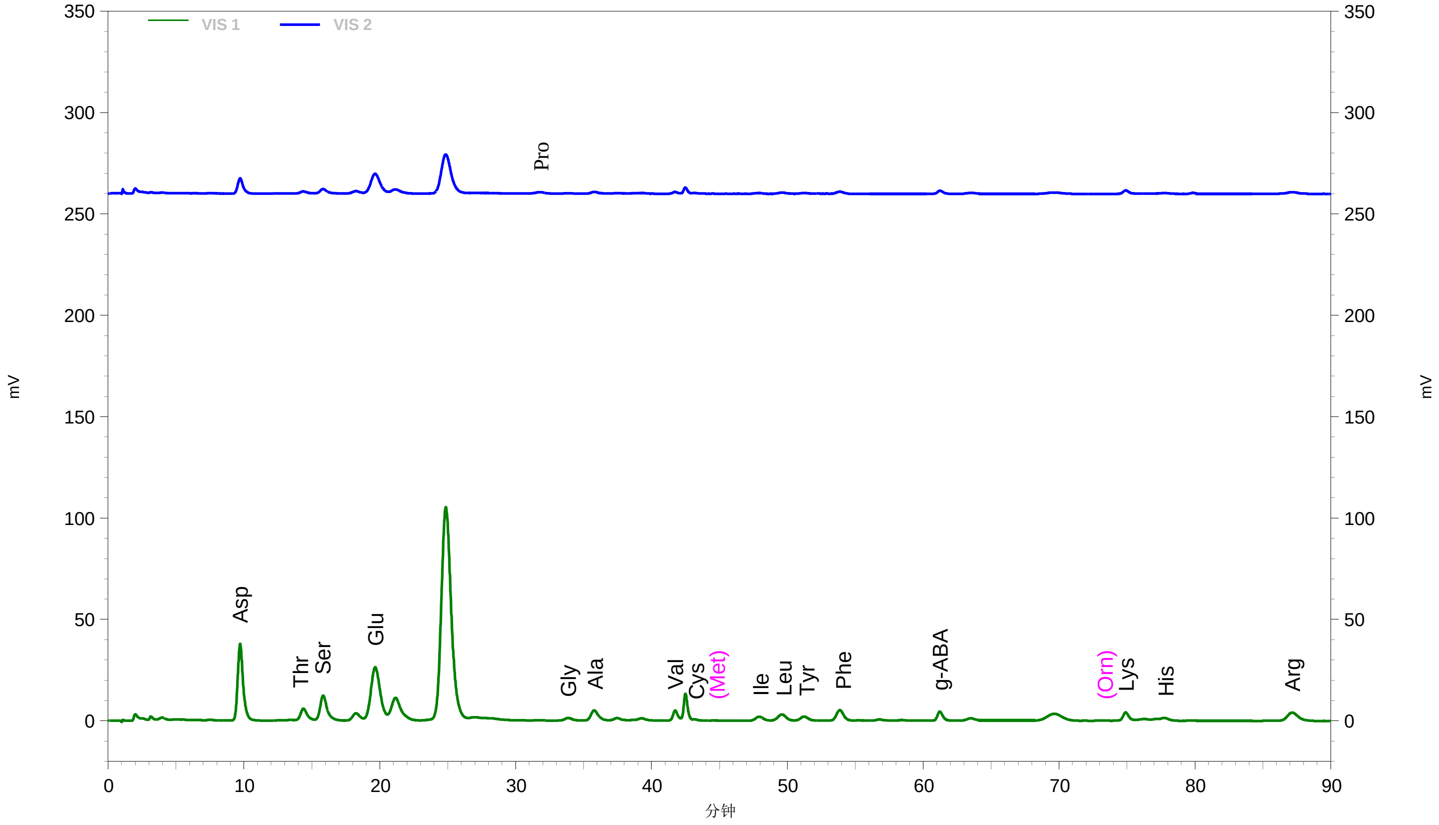

VIS 1 result

Peak #	Retention Time	Name	Area	Resolution	Mol concentration (nmol/mg)	CalcMol (ng/mg)
11	9.720	Asp	4208832	2.53	4.79	637.78
13	14.380	Thr	748685	2.50	0.82	97.17
14	15.833	Ser	1747096	1.65	1.89	199.09
16	19.660	Glu	5105589	1.21	5.67	833.69
20	33.867	Gly	199425	2.90	0.21	15.85
21	35.800	Ala	736064	1.93	0.77	68.31
24	41.740	Val	482808	3.06	0.48	56.77
26	43.160	Cys	52567	0.96	0.05	11.10
		Met			0.00 BDL	0.00
27	47.927	Ile	301044	5.01	0.29	38.33
28	49.607	Leu	495445	1.56	0.49	64.29
29	51.247	Tyr	296011	1.52	0.30	53.50
30	53.873	Phe	734246	2.62	0.75	123.39
34	61.233	g-ABA	491672	4.17	0.51	52.68
		Orn			0.00 BDL	0.00
38	74.907	Lys	500278	3.67	0.43	63.40
41	77.747	His	261679	0.00	0.24	37.16
42	87.167	Arg	893428	6.78	0.89	155.37
SUM			17254869		18.58	

VIS 2 result

Peak #	Retention Time	Name	Area	Resolution	Mol concentration (nmol/mg)	CalcMol (ng/mg)
8	31.840	Pro	86875	5.79	0.32	36.36
SUM			86875		0.32	
