# Supplementary material for: Dynamic Variation of Amino Acid Contents and Identification of Sterols in Xinyang Mao Jian Green Tea
Source: Molecules. 2022 Jun 1;27(11):3562. doi: 10.3390/molecules27113562 (PMC9182030; doi:10.3390/molecules27113562)
Supplement: Supplementary file 1 [file molecules-27-03562-s001.zip › pf-sm-chaye-yp202129670-sample-10-6ul-2021-7-3 5-58-11_REPORT-asn.pdf]

Hitachi Amino Acid Analyzer Report

Data File: E:\data\20210701 PF\PF-sm-chaye-yp202129670-sample-10-6ul-2021-7-3 5-58-11.dat
Method: E:\data\20210701 PF\sm-asn.met
Sample ID: {Sample ID}
Vial Number: 160 Inj. Volume(uL): 6

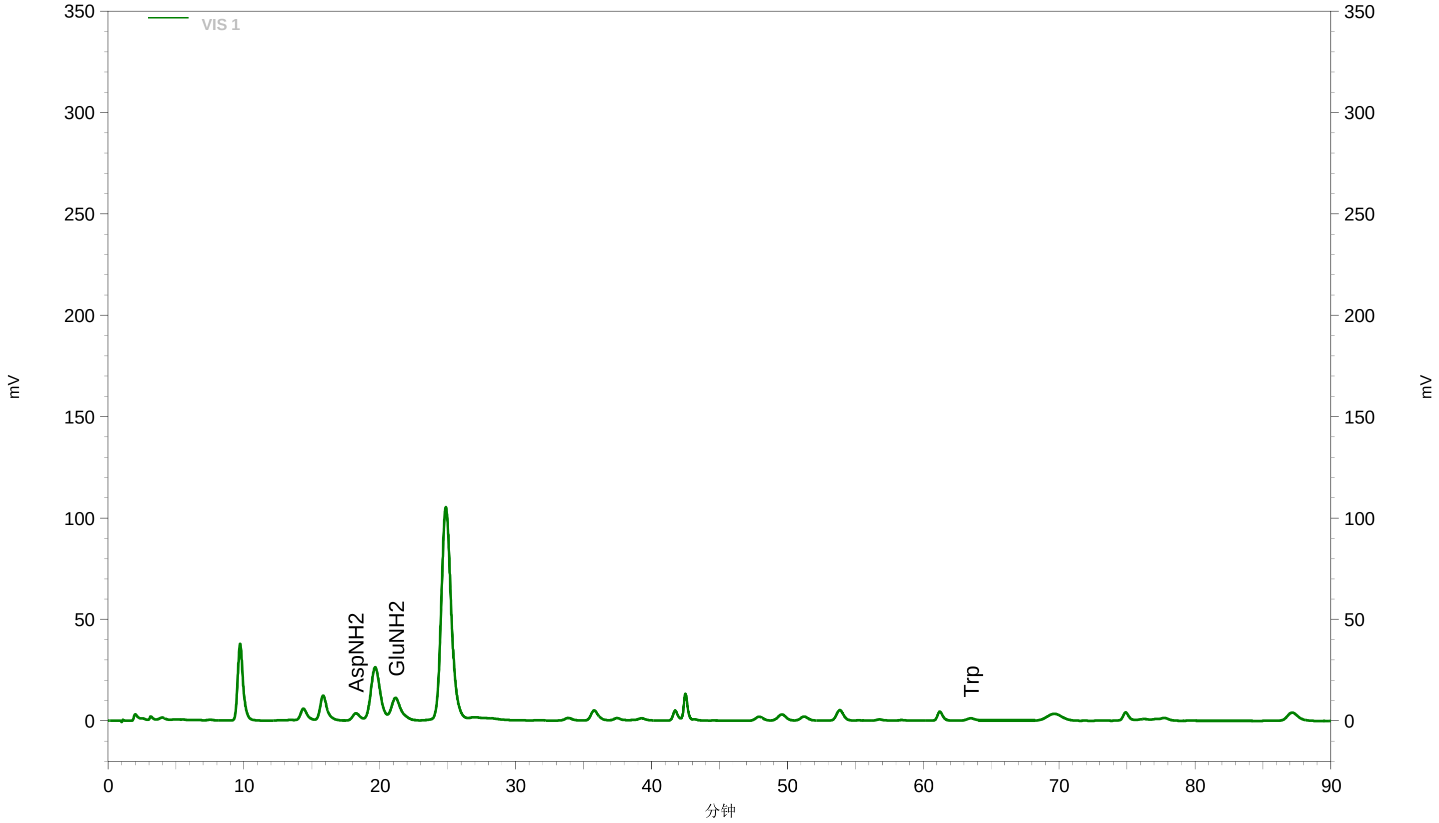

VIS 1 result

Peak #	Retention Time	Name	Area	Resolution	Mol concentration (nmol/mg)	CalcMol (ng/mg)
15	18.253	AspNH2	538534	2.51	0.75	99.46
17	21.167	GluNH2	2260069	1.16	2.76	403.73
35	63.513	Trp	192480	2.39	0.30	61.49

SUM			2991083		3.82	

VIS 2 result

Peak #	Retention Time	Name	Area	Resolution	Mol concentration (nmol/mg)	CalcMol (ng/mg)
