# Supplementary material for: Dynamic Variation of Amino Acid Contents and Identification of Sterols in Xinyang Mao Jian Green Tea
Source: Molecules. 2022 Jun 1;27(11):3562. doi: 10.3390/molecules27113562 (PMC9182030; doi:10.3390/molecules27113562)
Supplement: Supplementary file 1 [file molecules-27-03562-s001.zip › pf-sm-chaye-yp202129670-sample-11-6ul-2021-7-3 7-50-20_REPORT-.pdf]

Hitachi Amino Acid Analyzer Report

Data File: E:\data\20210701 PF\PF-sm-chaye-yp202129670-sample-11-6ul-2021-7-3 7-50-20.dat
Method: E:\data\20210701 PF\sm-chaye.met
Sample ID: {Sample ID}
Vial Number: 161 Inj. Volume(uL): 6

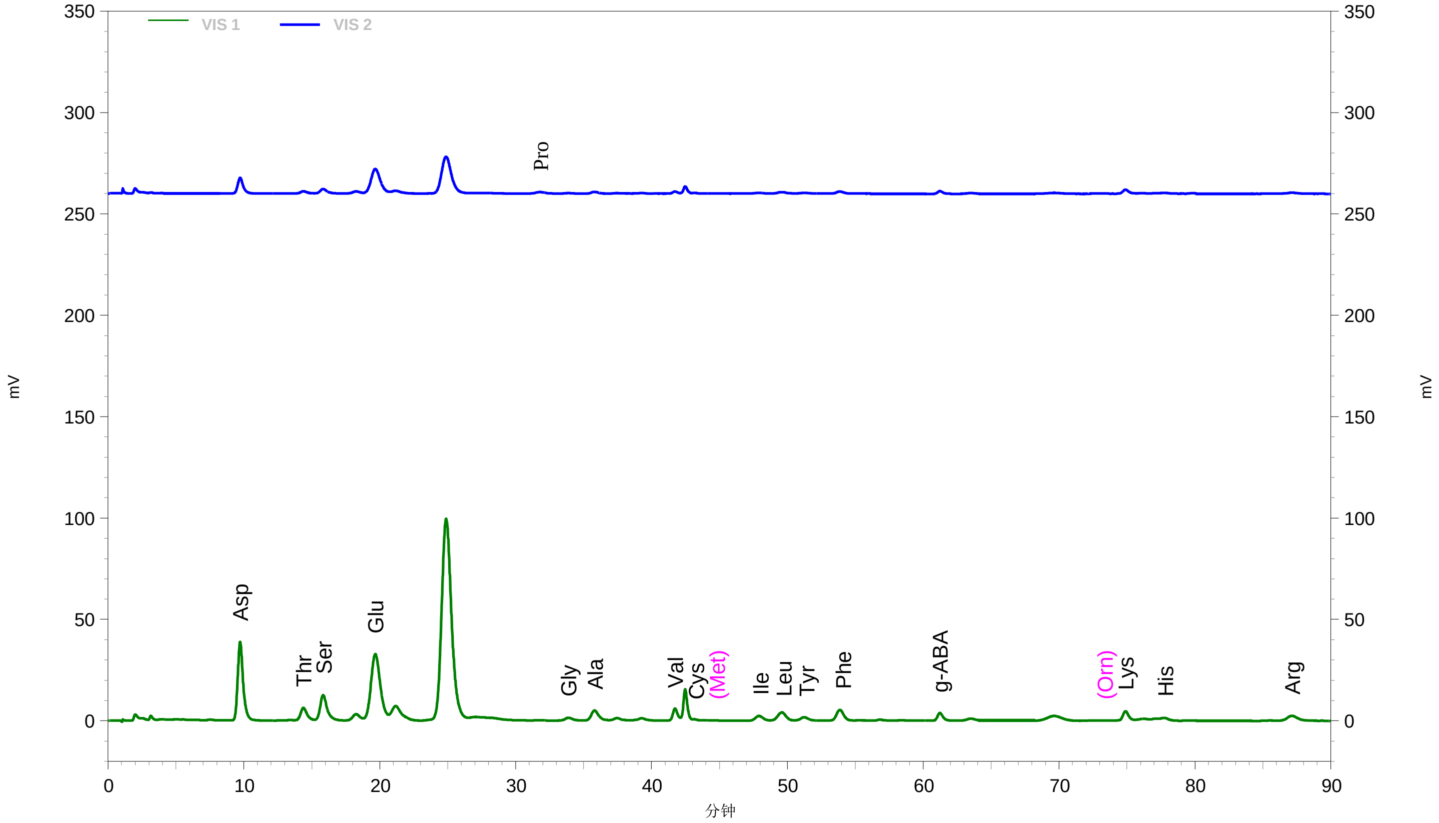

VIS 1 result

Peak #	Retention Time	Name	Area	Resolution	Mol concentration (nmol/mg)	CalcMol (ng/mg)
11	9.727	Asp	4298920	2.27	4.90	652.41
13	14.393	Thr	805949	2.38	0.88	104.76
14	15.840	Ser	1781452	1.65	1.93	203.31
16	19.680	Glu	6376402	1.22	7.09	1042.77
20	33.887	Gly	218884	2.79	0.23	17.43
21	35.807	Ala	735489	1.94	0.77	68.35
24	41.740	Val	574704	3.10	0.58	67.67
26	43.153	Cys	51318	0.90	0.05	10.86
		Met			0.00 BDL	0.00
27	47.927	Ile	358896	4.81	0.35	45.76
28	49.600	Leu	669637	1.55	0.66	87.02
29	51.247	Tyr	246766	1.51	0.25	44.67
30	53.867	Phe	749498	2.59	0.76	126.14
33	61.227	g-ABA	414520	4.44	0.43	44.48
		Orn			0.00 BDL	0.00
37	74.887	Lys	563154	3.68	0.49	71.48
40	77.740	His	221570	0.00	0.20	31.51
41	87.167	Arg	509628	6.62	0.51	88.76
SUM			18576787		20.08	

VIS 2 result

Peak #	Retention Time	Name	Area	Resolution	Mol concentration (nmol/mg)	CalcMol (ng/mg)
8	31.833	Pro	113733	5.61	0.41	47.67
SUM			113733		0.41	
