# Supplementary material for: Dynamic Variation of Amino Acid Contents and Identification of Sterols in Xinyang Mao Jian Green Tea
Source: Molecules. 2022 Jun 1;27(11):3562. doi: 10.3390/molecules27113562 (PMC9182030; doi:10.3390/molecules27113562)
Supplement: Supplementary file 1 [file molecules-27-03562-s001.zip › pf-sm-chaye-yp202129670-sample-12-6ul-2021-7-3 9-42-36_REPORT-.pdf]

Hitachi Amino Acid Analyzer Report

Data File: E:\data\20210701 PF\PF-sm-chaye-yp202129670-sample-12-6ul-2021-7-3 9-42-36.dat
Method: E:\data\20210701 PF\sm-chaye.met
Sample ID: {Sample ID}
Vial Number: 162 Inj. Volume(uL): 6

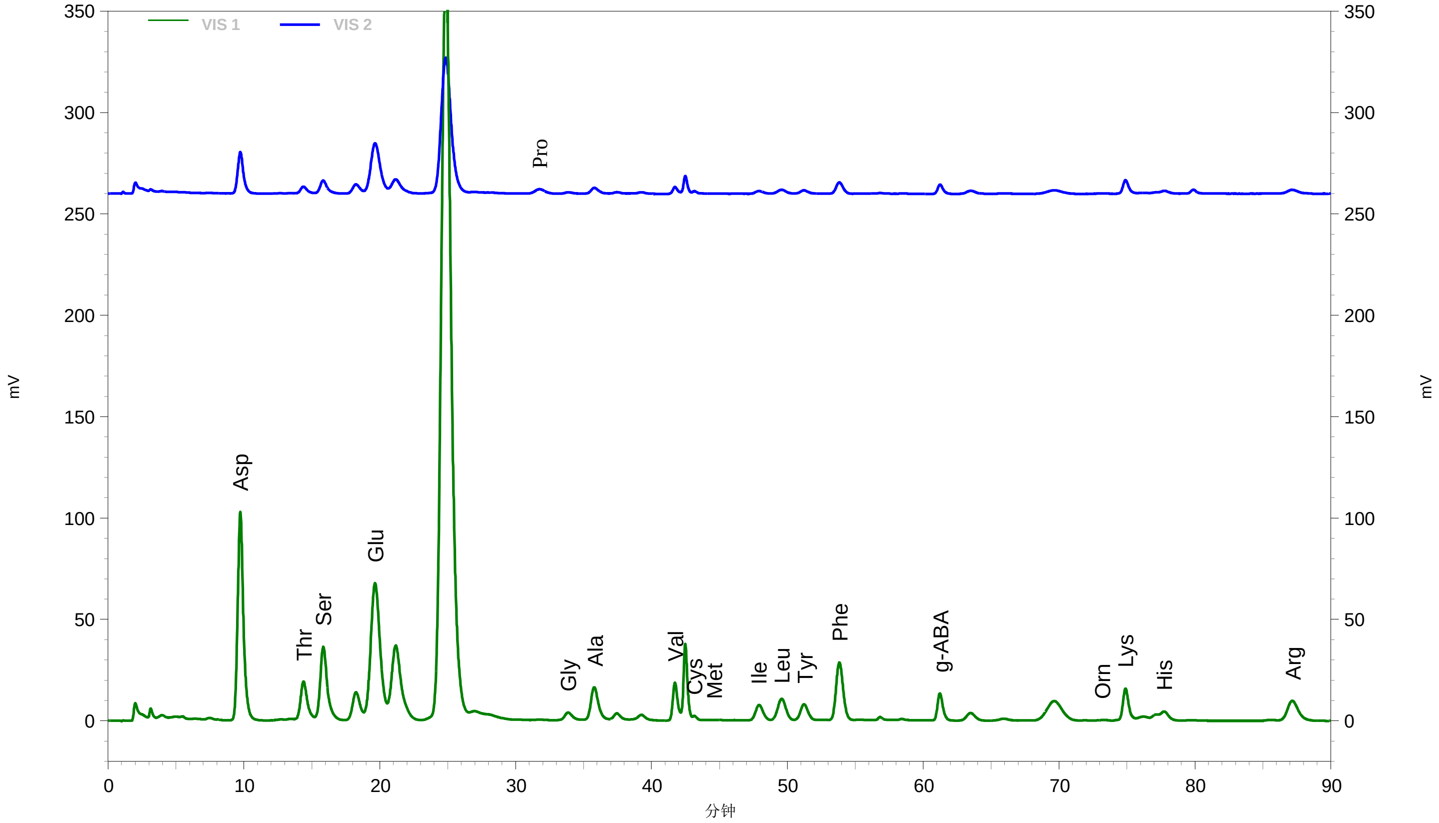

VIS 1 result

Peak #	Retention Time	Name	Area	Resolution	Mol concentration (nmol/mg)	CalcMol (ng/mg)
10	9.733	Asp	12096433	1.57	13.80	1836.68
13	14.387	Thr	2643197	0.80	2.89	343.74
14	15.847	Ser	5343692	1.61	5.81	610.16
16	19.660	Glu	13300112	1.18	14.79	2176.13
21	33.873	Gly	630790	1.95	0.67	50.25
22	35.793	Ala	2582244	1.86	2.70	240.11
25	41.733	Val	1825163	2.80	1.84	215.03
27	43.160	Cys	174976	0.95	0.15	37.03
28	44.567	Met	13033	1.59	0.01	1.90
29	47.927	Ile	1160694	3.45	1.13	148.07
30	49.587	Leu	1759868	1.53	1.74	228.82
31	51.233	Tyr	1182670	1.53	1.18	214.18
32	53.840	Phe	4116146	2.61	4.20	693.11
37	61.240	g-ABA	1475401	4.02	1.54	158.39
42	73.320	Orn	58839	1.53	0.04	5.51
43	74.893	Lys	1959790	1.62	1.70	248.86
46	77.733	His	761251	0.00	0.70	108.31
49	87.173	Arg	2165996	1.28	2.17	377.43
SUM			53250295		57.05	

VIS 2 result

Peak #	Retention Time	Name	Area	Resolution	Mol concentration (nmol/mg)	CalcMol (ng/mg)
8	31.747	Pro	393380	5.20	1.43	164.97
SUM			393380		1.43	
