# Supplementary material for: Dynamic Variation of Amino Acid Contents and Identification of Sterols in Xinyang Mao Jian Green Tea
Source: Molecules. 2022 Jun 1;27(11):3562. doi: 10.3390/molecules27113562 (PMC9182030; doi:10.3390/molecules27113562)
Supplement: Supplementary file 1 [file molecules-27-03562-s001.zip › pf-sm-chaye-yp202129670-sample-12-6ul-2021-7-3 9-42-36_REPORT-asn.pdf]

# Hitachi Amino Acid Analyzer Report

Data File: E:\data\20210701 PF\PF-sm-chaye-yp202129670-sample-12-6ul-2021-7-3 9-42-36.dat
Method: E:\data\20210701 PF\sm-asn.met
Sample ID: {Sample ID}
Vial Number: 162 Inj. Volume(uL): 6

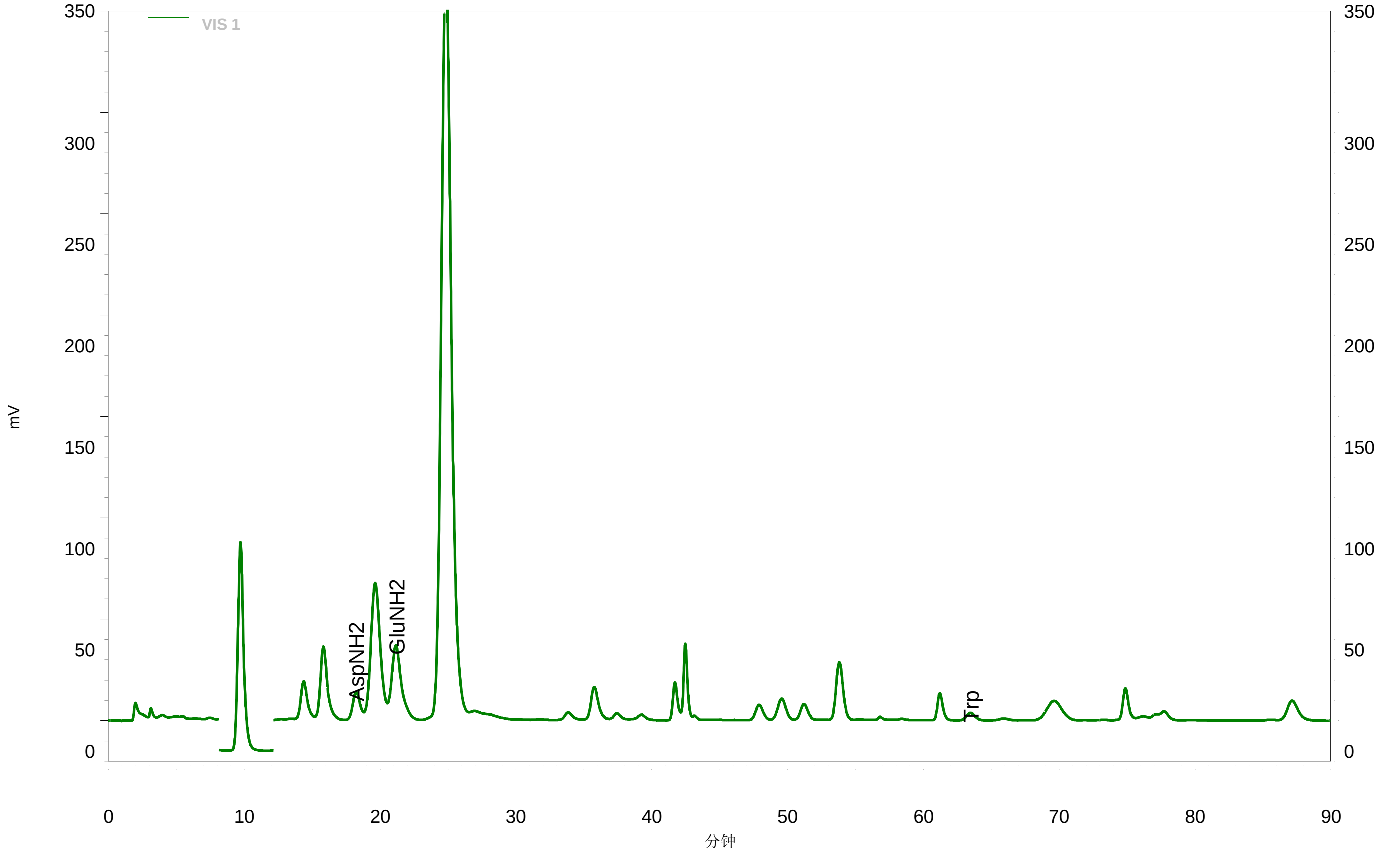


**VIS 1** result

| Peak # | Retention Time | Name | Area | Resolution | Mol concentration (nmol/mg) | CalcMol (ng/mg) |
|---|---|---|---|---|---|---|
| 15 | 18.260 | AspNH2 | 2109091 | 2.45 | 2.95 | 390.29 |
| 17 | 21.187 | GluNH2 | 7497620 | 1.17 | 9.19 | 1342.02 |
| 38 | 63.493 | Trp | 657679 | 2.35 | 1.03 | 210.53 |
| SUM | | | 10264390 | | 13.17 | |

**VIS 2** result

| Peak # | Retention Time | Name | Area | Resolution | Mol concentration (nmol/mg) | CalcMol (ng/mg) |
|---|---|---|---|---|---|---|
