# Supplementary material for: Dynamic Variation of Amino Acid Contents and Identification of Sterols in Xinyang Mao Jian Green Tea
Source: Molecules. 2022 Jun 1;27(11):3562. doi: 10.3390/molecules27113562 (PMC9182030; doi:10.3390/molecules27113562)
Supplement: Supplementary file 1 [file molecules-27-03562-s001.zip › pf-sm-chaye-yp202129670-sample-2-6ul-2021-7-2 9-23-14_REPORT-.pdf]

Hitachi Amino Acid Analyzer Report

Data File: E:\data\20210701 PF\PF-sm-chaye-yp202129670-sample-2-6ul-2021-7-2 9-23-14.dat
Method: E:\data\20210701 PF\sm-chaye.met
Sample ID: {Sample ID}
Vial Number: 152 Inj. Volume(uL): 6

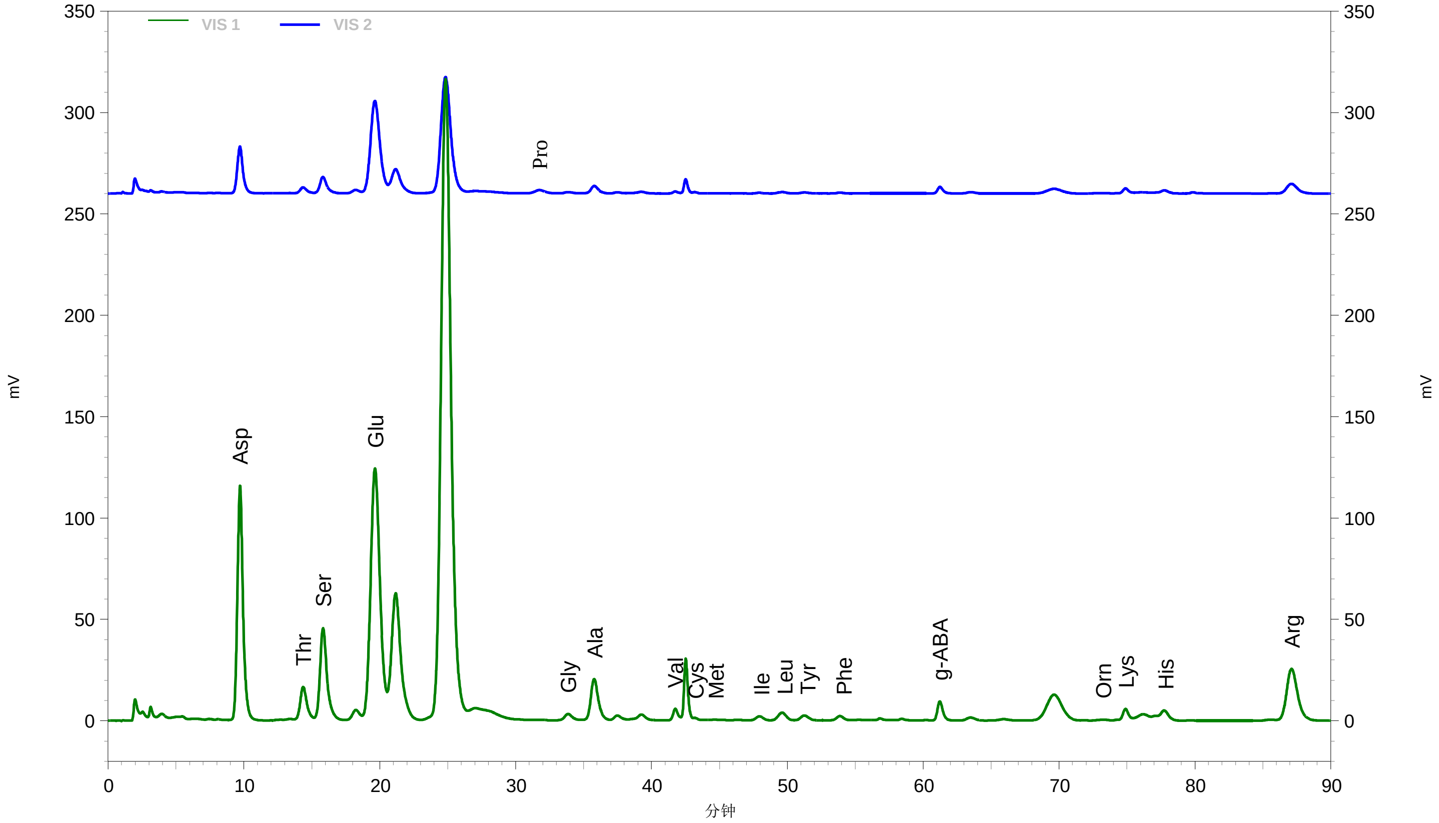

VIS 1 result

Peak #	Retention Time	Name	Area	Resolution	Mol concentration (nmol/mg)	CalcMol (ng/mg)
11	9.713	Asp	13438670	1.66	15.31	2037.42
13	14.360	Thr	2210892	1.05	2.41	287.09
14	15.827	Ser	6519553	1.64	7.07	743.31
16	19.647	Glu	24181552	1.19	26.86	3950.58
21	33.873	Gly	520627	1.85	0.55	41.41
22	35.793	Ala	3167028	1.87	3.30	294.04
25	41.753	Val	560141	2.75	0.56	65.89
27	43.167	Cys	87576	0.80	0.08	18.51
28	44.633	Met	9531	1.65	0.01	1.39
29	47.947	Ile	287361	3.96	0.28	36.60
30	49.620	Leu	627604	1.56	0.62	81.48
31	51.267	Tyr	377694	1.49	0.38	68.30
32	53.873	Phe	286324	2.55	0.29	48.14
37	61.233	g-ABA	1039843	3.99	1.08	111.46
41	73.280	Orn	87911	2.13	0.06	8.22
42	74.893	Lys	748638	1.55	0.65	94.92
44	77.740	His	1125177	0.93	1.03	159.85
46	87.113	Arg	5841462	0.94	5.83	1016.36
Sum			61117584		66.37	

VIS 2 result

Peak #	Retention Time	Name	Area	Resolution	Mol concentration (nmol/mg)	CalcMol (ng/mg)
8	31.747	Pro	280450	5.25	1.02	117.44
Sum			280450		1.02	
