# Supplementary material for: Dynamic Variation of Amino Acid Contents and Identification of Sterols in Xinyang Mao Jian Green Tea
Source: Molecules. 2022 Jun 1;27(11):3562. doi: 10.3390/molecules27113562 (PMC9182030; doi:10.3390/molecules27113562)
Supplement: Supplementary file 1 [file molecules-27-03562-s001.zip › pf-sm-chaye-yp202129670-sample-2-6ul-2021-7-2 9-23-14_REPORT-asn.pdf]

resultSum **Hitachi Amino Acid Analyzer Report**

Data File:	E:\data\20210701 PF\PF-sm-chaye-yp202129670-sample-2-6ul-2021-7-2 9-23-14.dat
Method:	E:\data\20210701 PF\sm-asn.met
Sample ID:	{Sample ID}
Vial Number:	152 Inj. Volume(uL): 6

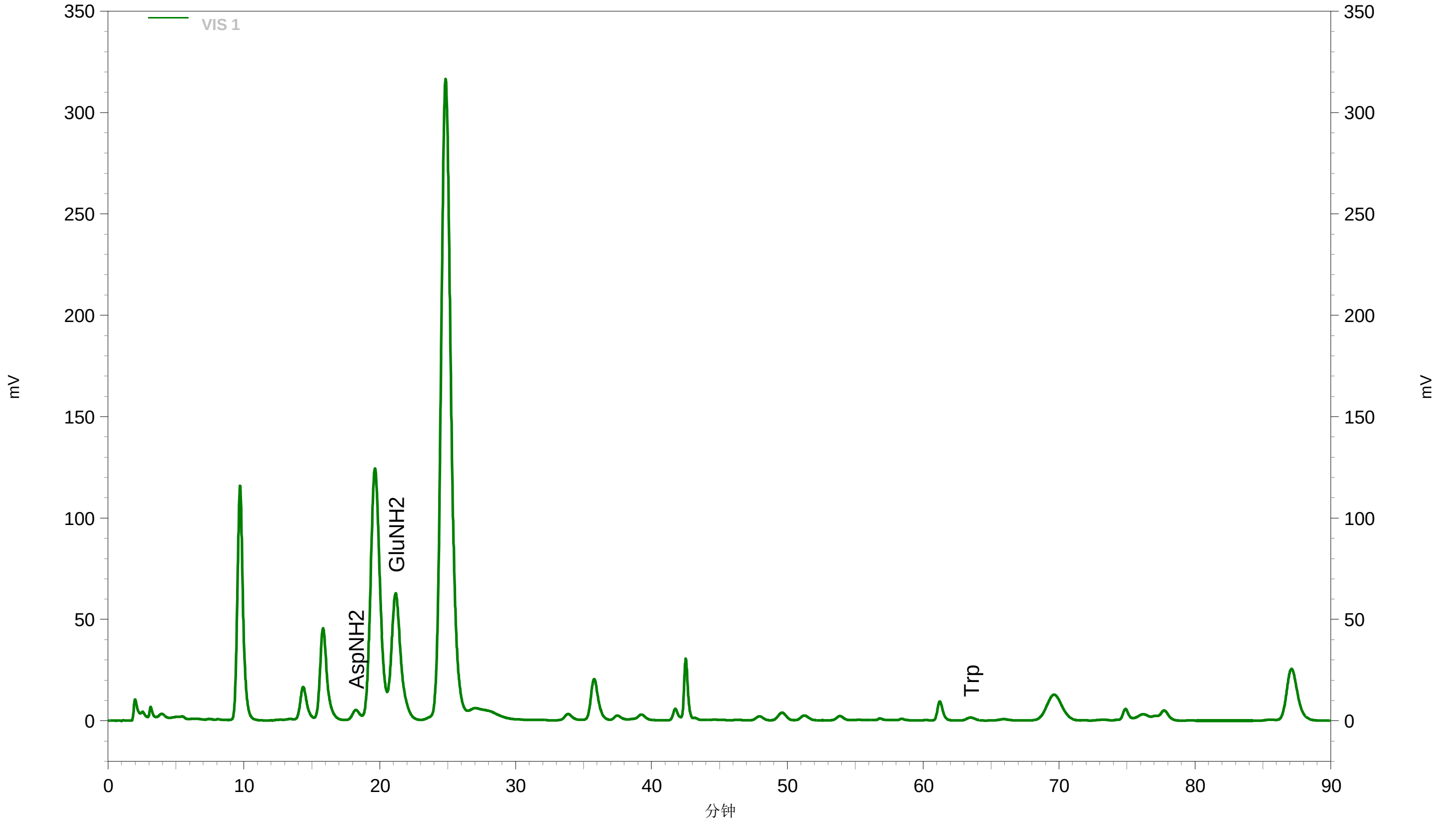

VIS 1 result

Peak #	Retention Time	Name	Area	Resolution	Mol concentration (nmol/mg)	CalcMol (ng/mg)
15	18.233	AspNH2	719087	2.45	1.01	132.87
17	21.180	GluNH2	12289147	1.19	15.03	2196.37
38	63.500	Trp	247036	2.40	0.39	78.96
Sum			13255270		16.43	

VIS 2 result

Peak #	Retention Time	Name	Area	Resolution	Mol concentration (nmol/mg)	CalcMol (ng/mg)
