# Supplementary material for: Dynamic Variation of Amino Acid Contents and Identification of Sterols in Xinyang Mao Jian Green Tea
Source: Molecules. 2022 Jun 1;27(11):3562. doi: 10.3390/molecules27113562 (PMC9182030; doi:10.3390/molecules27113562)
Supplement: Supplementary file 1 [file molecules-27-03562-s001.zip › pf-sm-chaye-yp202129670-sample-3-6ul-2021-7-2 11-15-26_REPORT-.pdf]

eryHitachi Amino Acid Analyzer Report

Data File: E:\data\20210701 PF\PF-sm-chaye-yp202129670-sample-3-6ul-2021-7-2 11-15-26.dat
Method: E:\data\20210701 PF\sm-chaye.met
Sample ID: {Sample ID}
Vial Number: 153 Inj. Volume(uL): 6

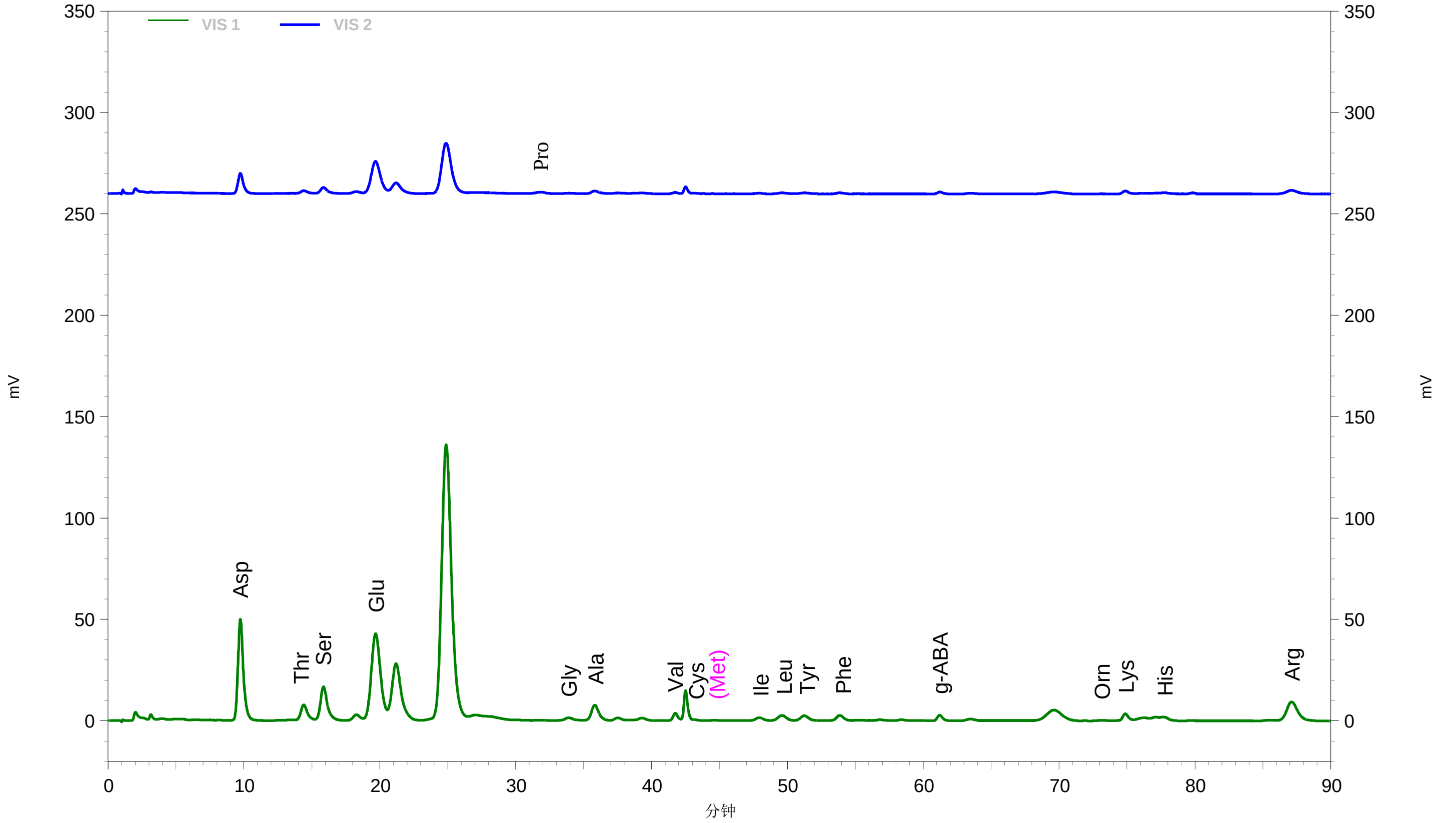

VIS 1 result

Peak #	Retention Time	Name	Area	Resolution	Mol concentration (nmol/mg)	CalcMol (ng/mg)
10	9.740	Asp	5553329	2.48	6.34	843.20
12	14.400	Thr	1022819	1.06	1.12	133.01
13	15.860	Ser	2371951	1.65	2.58	270.84
15	19.693	Glu	8246905	1.23	9.17	1349.34
19	33.907	Gly	197470	2.84	0.21	15.73
20	35.827	Ala	1103832	1.95	1.15	102.64
23	41.747	Val	355229	2.98	0.36	41.85
25	43.140	Cys	36104	0.74	0.03	7.64
		Met			0.00 BDL	0.00
26	47.940	Ile	219649	4.30	0.21	28.02
27	49.620	Leu	427821	1.57	0.42	55.63
28	51.253	Tyr	374281	1.48	0.37	67.78
29	53.873	Phe	363686	2.56	0.37	61.24
34	61.220	g-ABA	301350	4.08	0.31	32.35
38	73.187	Orn	41189	2.16	0.03	3.85
39	74.880	Lys	428956	1.71	0.37	54.47
42	77.687	His	308287	0.29	0.28	43.86
43	87.120	Arg	2051377	5.92	2.05	357.46
Sum			23404235		25.39	

VIS 2 result

Peak #	Retention Time	Name	Area	Resolution	Mol concentration (nmol/mg)	CalcMol (ng/mg)
8	31.833	Pro	99599	5.71	0.36	41.77
Sum			99599		0.36	
