# Supplementary material for: Dynamic Variation of Amino Acid Contents and Identification of Sterols in Xinyang Mao Jian Green Tea
Source: Molecules. 2022 Jun 1;27(11):3562. doi: 10.3390/molecules27113562 (PMC9182030; doi:10.3390/molecules27113562)
Supplement: Supplementary file 1 [file molecules-27-03562-s001.zip › pf-sm-chaye-yp202129670-sample-3-6ul-2021-7-2 11-15-26_REPORT-asn.pdf]

Hitachi Amino Acid Analyzer Report

Data File: E:\data\20210701 PF\PF-sm-chaye-yp202129670-sample-3-6ul-2021-7-2 11-15-26.dat
Method: E:\data\20210701 PF\sm-asn.met
Sample ID: {Sample ID}
Vial Number: 153 Inj. Volume(uL): 6

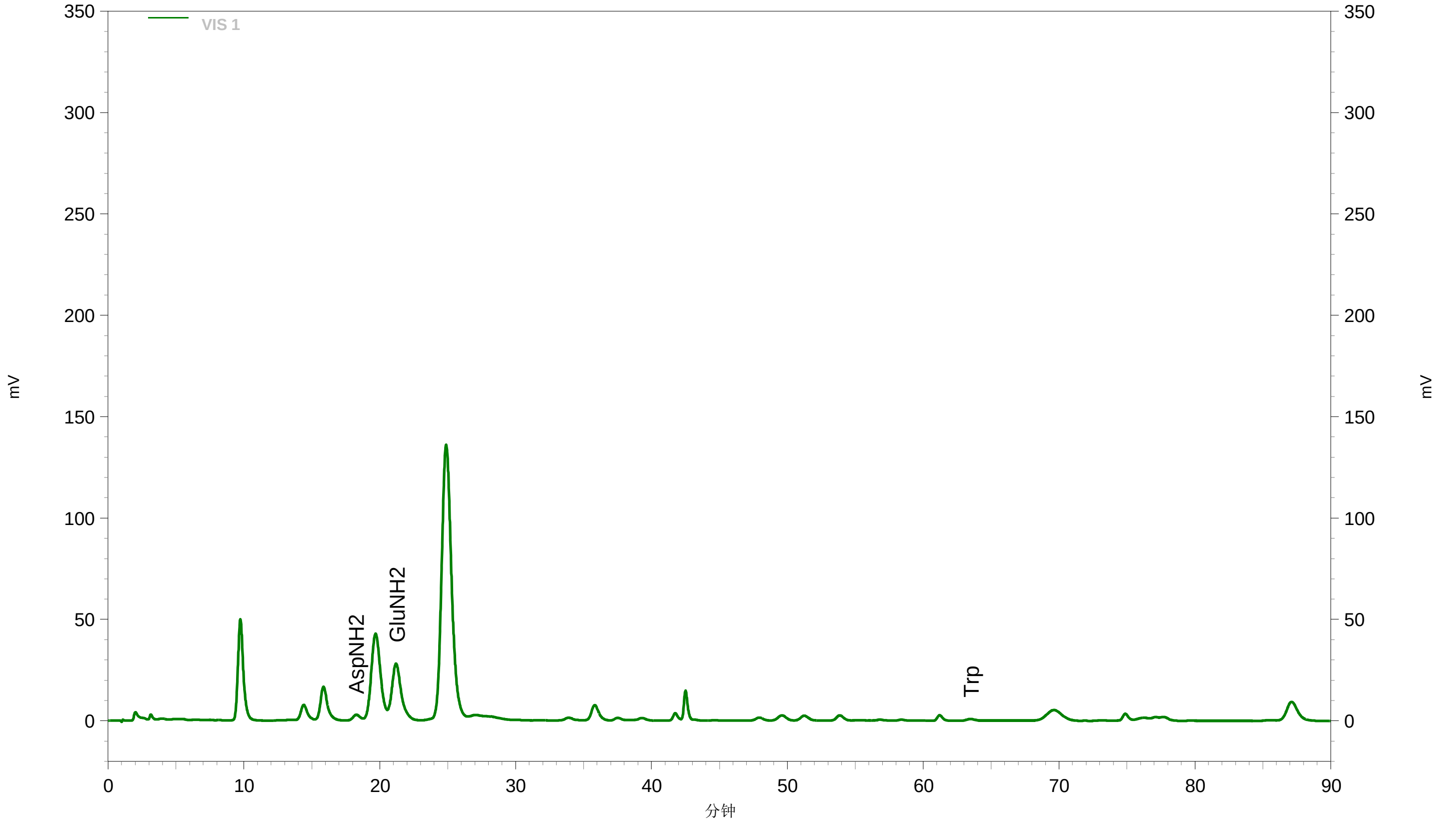

VIS 1 result

Peak #	Retention Time	Name	Area	Resolution	Mol concentration (nmol/mg)	CalcMol (ng/mg)
14	18.267	AspNH2	413410	2.49	0.58	76.50
16	21.207	GluNH2	5427261	1.20	6.65	971.44
35	63.507	Trp	143486	2.44	0.22	45.93
Sum			5984157		7.45	

VIS 2 result

Peak #	Retention Time	Name	Area	Resolution	Mol concentration (nmol/mg)	CalcMol (ng/mg)
