# Supplementary material for: Dynamic Variation of Amino Acid Contents and Identification of Sterols in Xinyang Mao Jian Green Tea
Source: Molecules. 2022 Jun 1;27(11):3562. doi: 10.3390/molecules27113562 (PMC9182030; doi:10.3390/molecules27113562)
Supplement: Supplementary file 1 [file molecules-27-03562-s001.zip › pf-sm-chaye-yp202129670-sample-4-6ul-2021-7-2 13-07-43_REPORT-.pdf]

umHitachi Amino Acid Analyzer Report

Data File: E:\data\20210701 PF\PF-sm-chaye-yp202129670-sample-4-6ul-2021-7-2 13-07-43.dat
Method: E:\data\20210701 PF\sm-chaye.met
Sample ID: {Sample ID}
Vial Number: 154 Inj. Volume(uL): 6

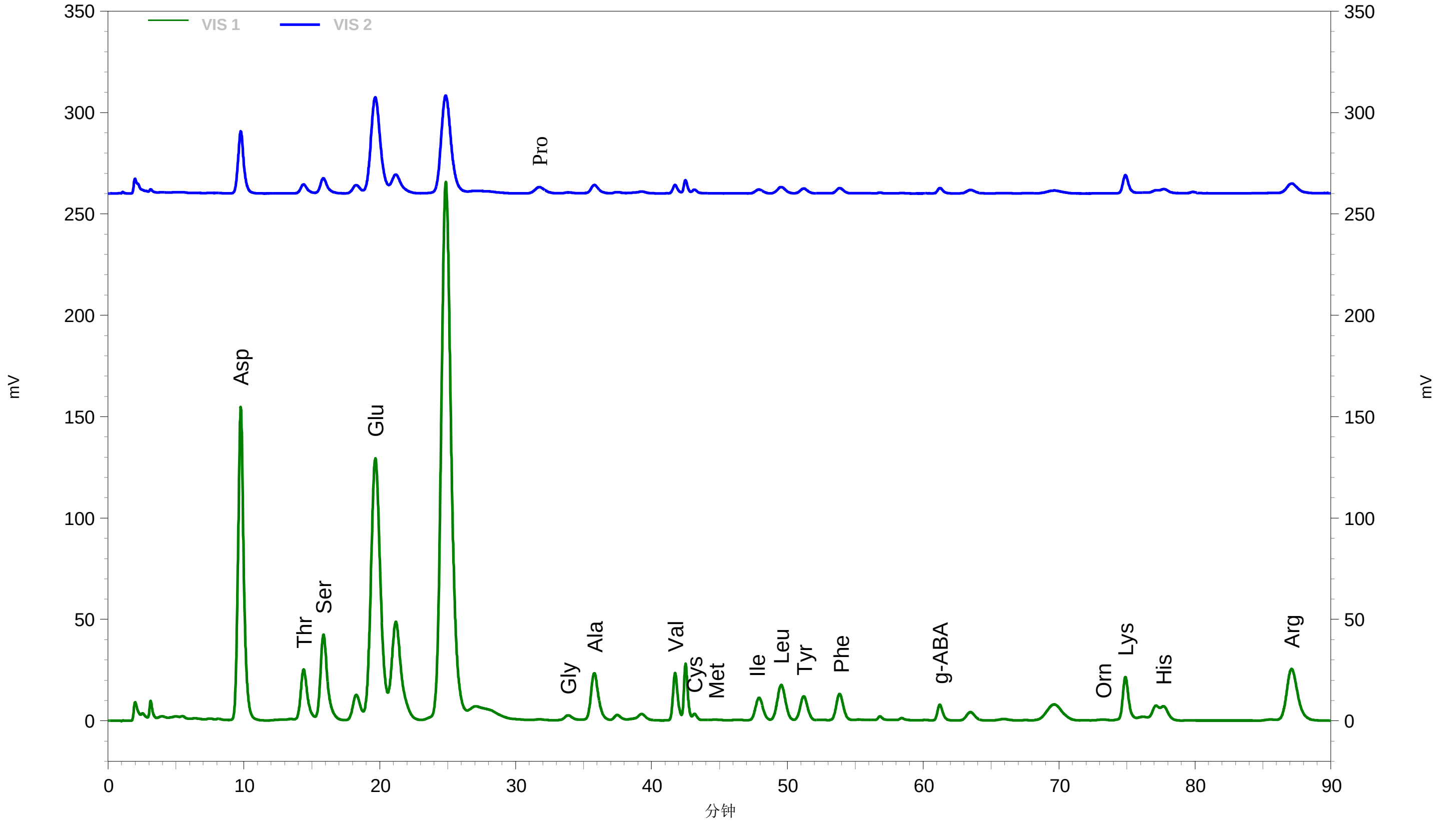

VIS 1 result

Peak #	Retention Time	Name	Area	Resolution	Mol concentration (nmol/mg)	CalcMol (ng/mg)
11	9.767	Asp	18148749	1.76	20.68	2752.88
14	14.400	Thr	3450608	0.83	3.76	448.29
15	15.853	Ser	6144424	1.61	6.67	700.89
17	19.673	Glu	25328141	1.18	28.14	4139.97
22	33.873	Gly	342202	1.83	0.36	27.23
23	35.800	Ala	3479309	1.94	3.63	323.20
26	41.740	Val	2260862	2.75	2.27	266.10
28	43.167	Cys	274723	1.11	0.24	58.09
29	44.720	Met	24998	1.94	0.02	3.64
30	47.920	Ile	1724353	3.27	1.67	219.75
31	49.560	Leu	2939387	1.50	2.91	381.80
32	51.220	Tyr	1794134	1.54	1.79	324.59
33	53.853	Phe	1847170	2.65	1.88	310.73
39	61.233	g-ABA	848534	1.50	0.88	91.00
44	73.240	Orn	80985	1.38	0.06	7.57
45	74.887	Lys	2662216	1.57	2.31	337.72
48	77.693	His	1085129	0.27	0.99	154.24
50	87.120	Arg	5807124	1.00	5.80	1010.89
Sum			78243048		84.09	

VIS 2 result

Peak #	Retention Time	Name	Area	Resolution	Mol concentration (nmol/mg)	CalcMol (ng/mg)
8	31.747	Pro	586388	5.19	2.13	245.67
Sum			586388		2.13	
