# Supplementary material for: Dynamic Variation of Amino Acid Contents and Identification of Sterols in Xinyang Mao Jian Green Tea
Source: Molecules. 2022 Jun 1;27(11):3562. doi: 10.3390/molecules27113562 (PMC9182030; doi:10.3390/molecules27113562)
Supplement: Supplementary file 1 [file molecules-27-03562-s001.zip › pf-sm-chaye-yp202129670-sample-5-6ul-2021-7-2 14-59-59_REPORT-.pdf]

Hitachi Amino Acid Analyzer Report

Data File: E:\data\20210701 PF\PF-sm-chaye-yp202129670-sample-5-6ul-2021-7-2 14-59-59.dat
Method: E:\data\20210701 PF\sm-chaye.met
Sample ID: {Sample ID}
Vial Number: 155 Inj. Volume(uL): 6

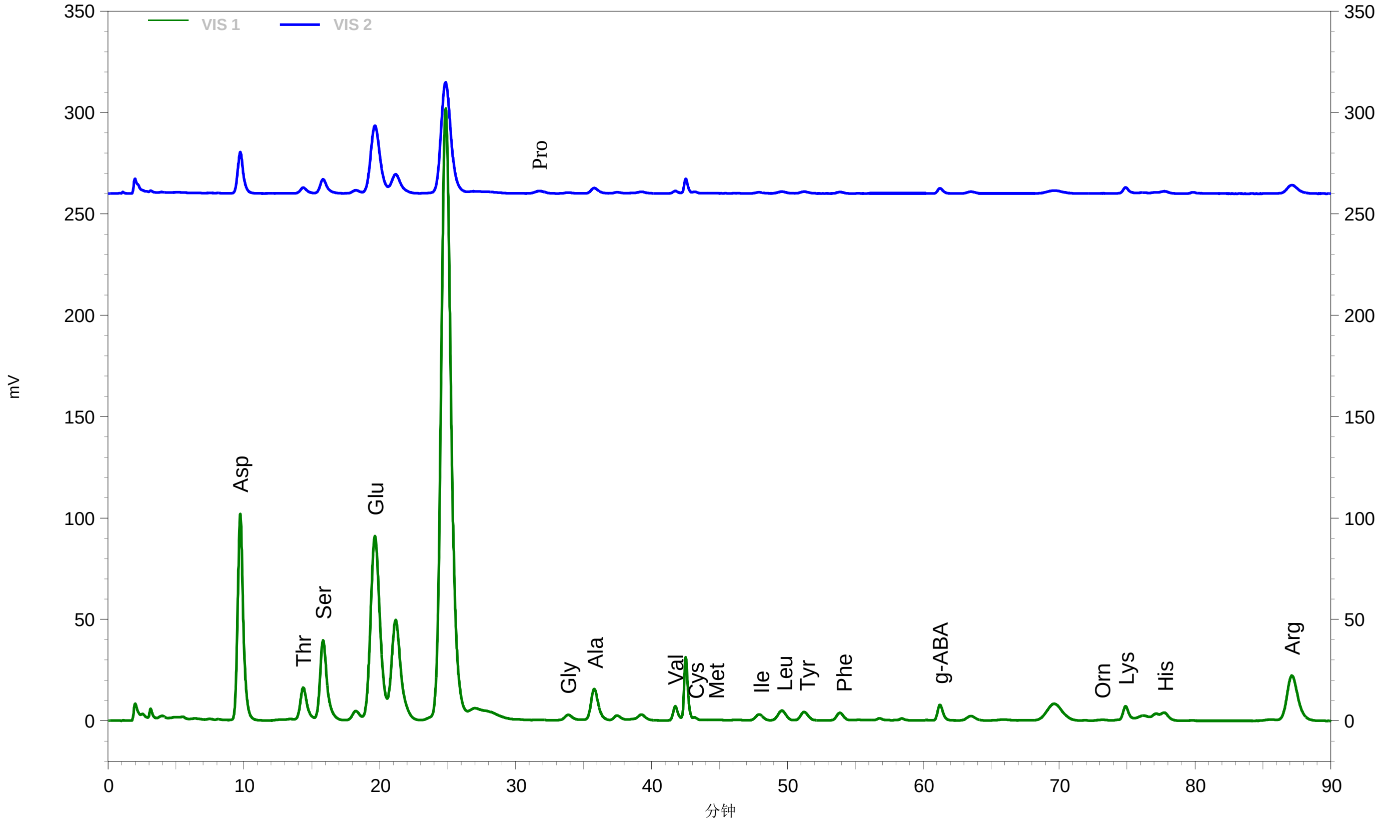

VIS 1 result

Peak #	Retention Time	Name	Area	Resolution	Mol concentration (nmol/mg)	CalcMol (ng/mg)
11	9.733	Asp	11827966	1.71	13.50	1796.81
14	14.380	Thr	2218530	0.89	2.42	288.66
15	15.840	Ser	5729836	1.62	6.23	654.58
17	19.653	Glu	17685942	1.19	19.68	2895.17
22	33.873	Gly	381375	0.00	0.40	30.40
23	35.793	Ala	2266203	1.94	2.37	210.83
26	41.753	Val	684728	2.80	0.69	80.71
28	43.167	Cys	109178	0.88	0.10	23.12
29	44.680	Met	13725	1.80	0.01	2.00
30	47.940	Ile	439443	3.75	0.43	56.09
31	49.600	Leu	797699	1.54	0.79	103.77
32	51.247	Tyr	644946	1.50	0.64	116.86
33	53.880	Phe	517940	2.60	0.53	87.26
39	61.233	g-ABA	847308	0.00	0.88	91.01
43	73.213	Orn	71159	2.12	0.05	6.66
44	74.900	Lys	923266	1.67	0.80	117.30
47	77.733	His	662889	0.29	0.61	94.36
49	87.140	Arg	5079691	0.99	5.08	885.59
Sum			50901824		55.22	

VIS 2 result

Peak #	Retention Time	Name	Area	Resolution	Mol concentration (nmol/mg)	CalcMol (ng/mg)
8	31.727	Pro	211679	5.25	0.77	88.82
Sum			211679		0.77	
