# Supplementary material for: Dynamic Variation of Amino Acid Contents and Identification of Sterols in Xinyang Mao Jian Green Tea
Source: Molecules. 2022 Jun 1;27(11):3562. doi: 10.3390/molecules27113562 (PMC9182030; doi:10.3390/molecules27113562)
Supplement: Supplementary file 1 [file molecules-27-03562-s001.zip › pf-sm-chaye-yp202129670-sample-6-6ul-2021-7-2 16-52-14_REPORT-.pdf]

Hitachi Amino Acid Analyzer Report

Data File: E:\data\20210701 PF\PF-sm-chaye-yp202129670-sample-6-6ul-2021-7-2 16-52-14.dat
Method: E:\data\20210701 PF\sm-chaye.met
Sample ID: {Sample ID}
Vial Number: 156 Inj. Volume(uL): 6

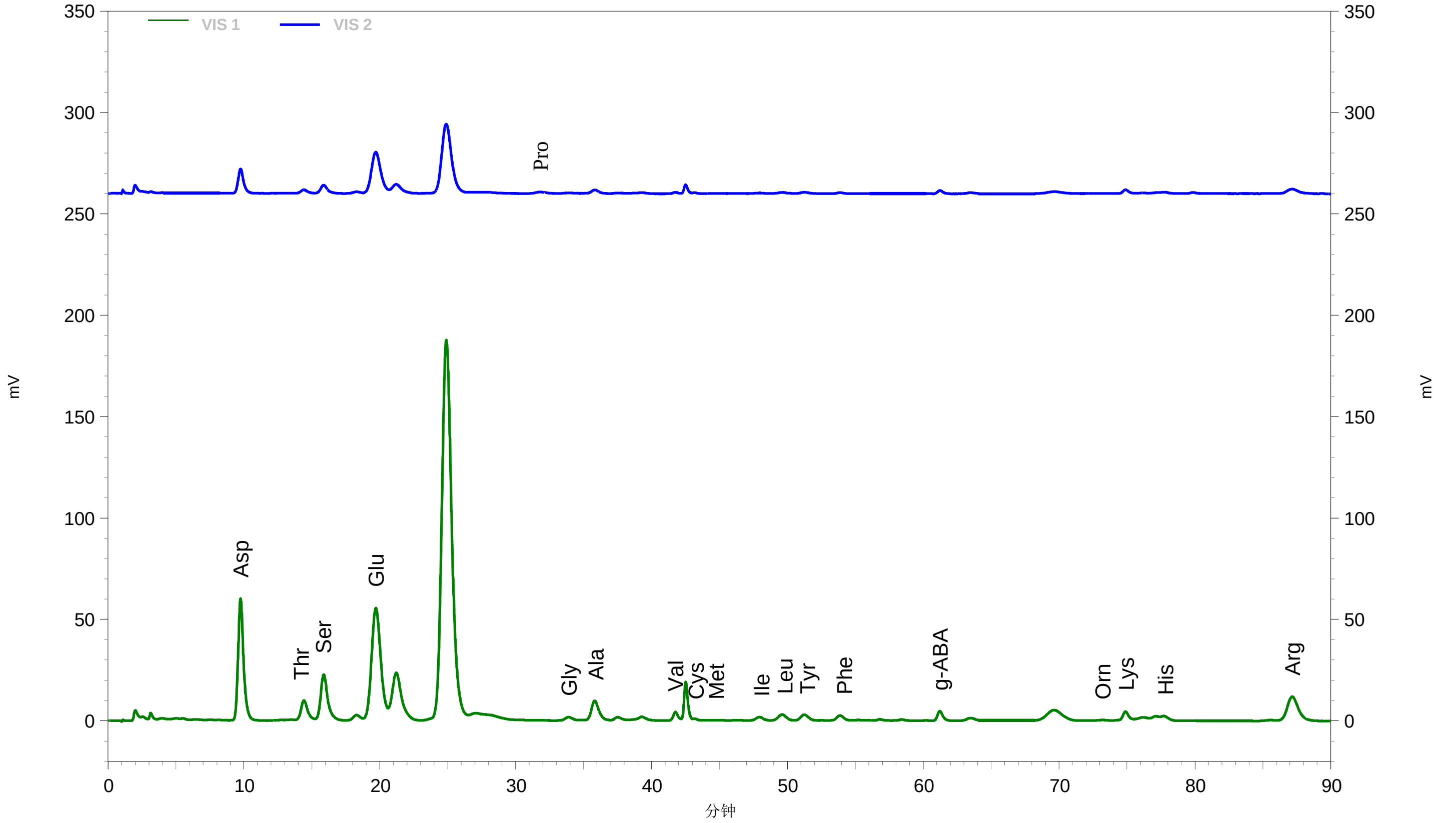

VIS 1 result

Peak #	Retention Time	Name	Area	Resolution	Mol concentration (nmol/mg)	CalcMol (ng/mg)
11	9.753	Asp	6749628	2.28	7.68	1022.79
13	14.420	Thr	1310334	1.09	1.43	170.06
14	15.873	Ser	3262846	1.63	3.54	371.82
16	19.713	Glu	10733478	1.22	11.91	1752.67
20	33.920	Gly	239121	2.81	0.25	19.01
21	35.833	Ala	1432720	1.94	1.49	132.95
24	41.760	Val	406067	2.75	0.41	47.75
26	43.167	Cys	71192	0.92	0.06	15.04
27	44.673	Met	4717	0.00	0.00	0.69
28	47.967	Ile	246694	0.00	0.24	31.41
29	49.633	Leu	479274	1.55	0.47	62.19
30	51.253	Tyr	445646	1.48	0.44	80.54
31	53.893	Phe	337667	2.62	0.34	56.75
36	61.233	g-ABA	512740	4.07	0.53	54.93
40	73.193	Orn	44164	2.14	0.03	4.13
41	74.887	Lys	564791	1.70	0.49	71.58
44	77.720	His	368086	0.30	0.34	52.27
46	87.167	Arg	2723840	1.04	2.72	473.69
Sum			29933005		32.40	

VIS 2 result

Peak #	Retention Time	Name	Area	Resolution	Mol concentration (nmol/mg)	CalcMol (ng/mg)
8	31.807	Pro	123031	5.42	0.45	51.49
Sum			123031		0.45	
