# Supplementary material for: Dynamic Variation of Amino Acid Contents and Identification of Sterols in Xinyang Mao Jian Green Tea
Source: Molecules. 2022 Jun 1;27(11):3562. doi: 10.3390/molecules27113562 (PMC9182030; doi:10.3390/molecules27113562)
Supplement: Supplementary file 1 [file molecules-27-03562-s001.zip › PF-sm-chaye-yp202129670-sample-7-6ul-2021-7-3 0-21-13.pdf]

Hitachi Amino Acid Analyzer Report

Data File: E:\data\20210701 PF\PF-sm-chaye-yp202129670-sample-7-6ul-2021-7-3 0-21-13.dat
Method: E:\data\20210701 PF\sm-chaye.met
Sample ID: {Sample ID}
Vial Number: 157 Inj. Volume(uL): 6

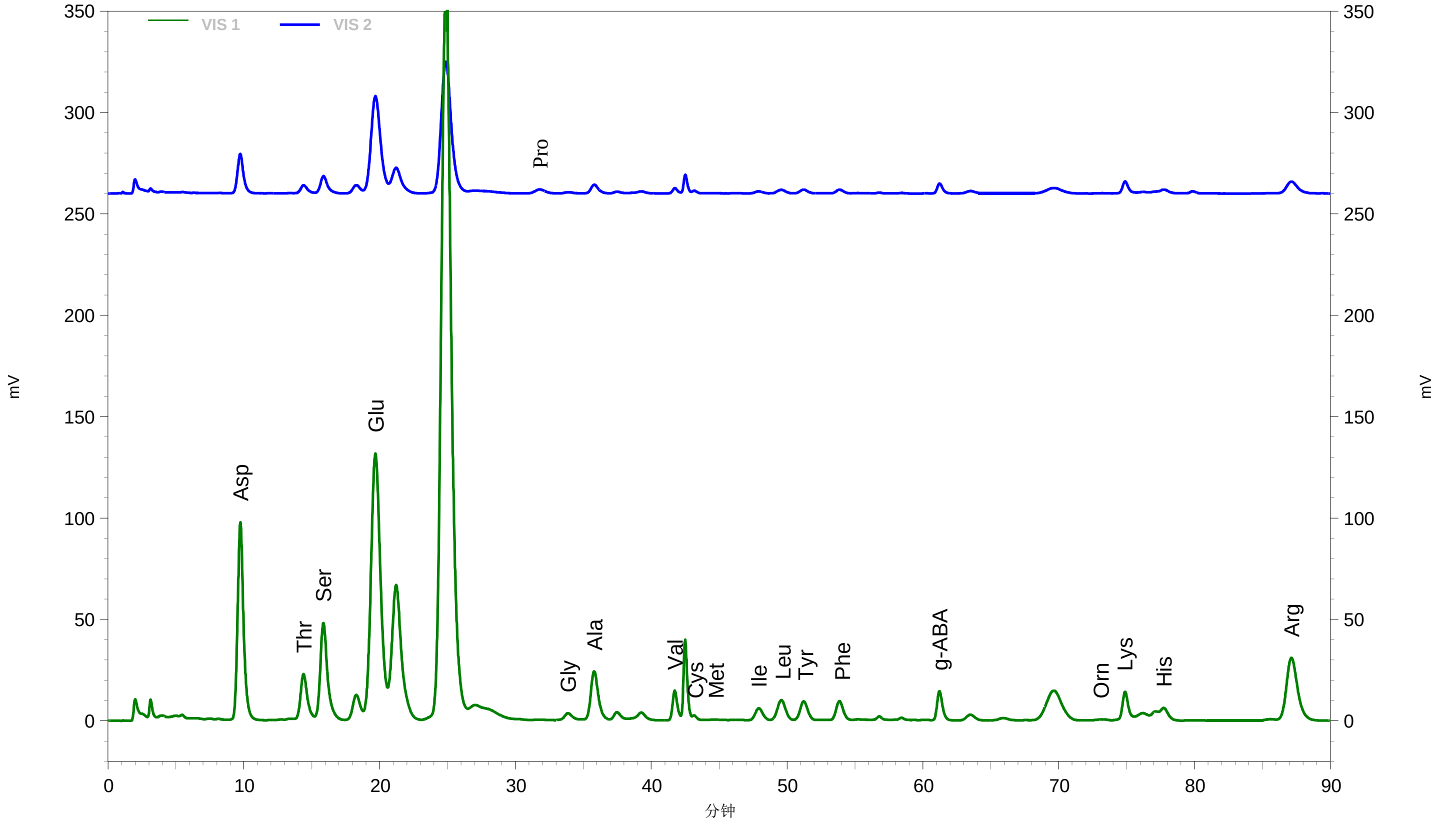

VIS 1 result

Peak #	Retention Time	Name	Area	Resolution	Mol concentration (nmol/mg)	CalcMol (ng/mg)
10	9.747	Asp	11939098	1.64	13.60	1810.07
13	14.407	Thr	3202398	0.84	3.49	415.84
14	15.867	Ser	7164672	1.58	7.77	816.86
16	19.693	Glu	26043071	1.17	28.92	4254.70
21	33.880	Gly	491291	1.97	0.52	39.08
22	35.807	Ala	3608270	1.92	3.76	335.01
25	41.740	Val	1420389	2.75	1.43	167.09
27	43.160	Cys	186119	0.96	0.16	39.33
28	44.673	Met	15355	1.93	0.01	2.23
30	47.927	Ile	910701	0.00	0.88	116.00
31	49.580	Leu	1653569	1.52	1.64	214.68
32	51.227	Tyr	1422120	1.51	1.42	257.16
33	53.867	Phe	1332768	2.62	1.36	224.08
39	61.227	g-ABA	1579970	1.48	1.64	169.36
44	73.240	Orn	120374	1.34	0.09	11.25
45	74.893	Lys	1793205	1.54	1.56	227.37
48	77.733	His	1057782	0.00	0.97	150.28
50	87.140	Arg	7115823	0.96	7.11	1238.09
Sum			71056975		76.33	

VIS 2 result

Peak #	Retention Time	Name	Area	Resolution	Mol concentration (nmol/mg)	CalcMol (ng/mg)
8	31.813	Pro	372603	5.19	1.36	156.03
Sum			372603		1.36	
