# Supplementary material for: Dynamic Variation of Amino Acid Contents and Identification of Sterols in Xinyang Mao Jian Green Tea
Source: Molecules. 2022 Jun 1;27(11):3562. doi: 10.3390/molecules27113562 (PMC9182030; doi:10.3390/molecules27113562)
Supplement: Supplementary file 1 [file molecules-27-03562-s001.zip › pf-sm-chaye-yp202129670-sample-7-6ul-2021-7-3 0-21-13_REPORT-asn.pdf]

Hitachi Amino Acid Analyzer Report

Data File: E:\data\20210701 PF\PF-sm-chaye-yp202129670-sample-7-6ul-2021-7-3 0-21-13.dat
Method: E:\data\20210701 PF\sm-asn.met
Sample ID: {Sample ID}
Vial Number: 157 Inj. Volume(uL): 6

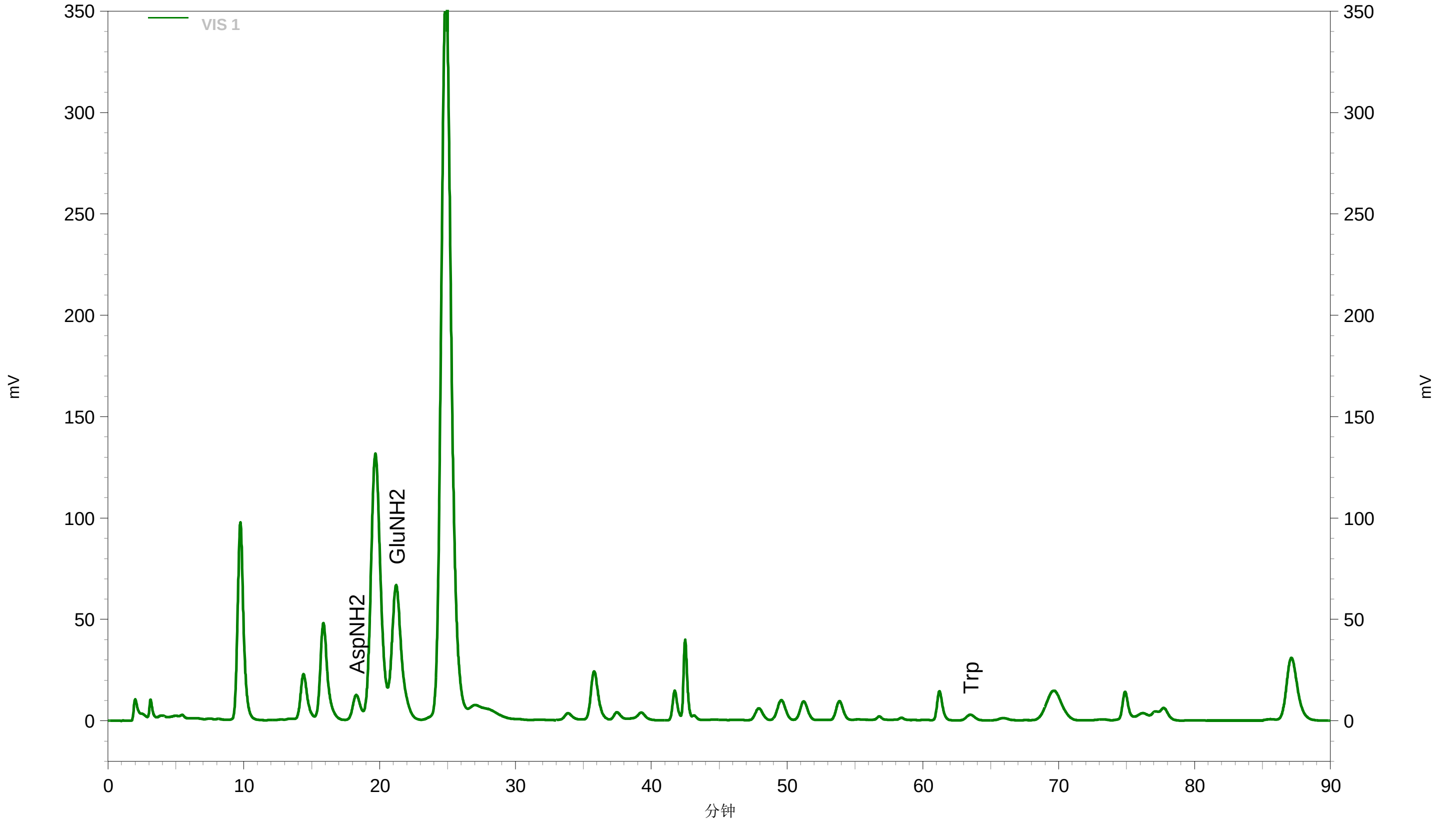

VIS 1 result

Peak #	Retention Time	Name	Area	Resolution	Mol concentration (nmol/mg)	CalcMol (ng/mg)
15	18.287	AspNH2	1893684	2.40	2.65	349.90
17	21.227	GluNH2	13330735	1.17	16.31	2382.53
40	63.500	Trp	493274	2.38	0.77	157.66

Sum			15717693		19.73	

VIS 2 result

Peak #	Retention Time	Name	Area	Resolution	Mol concentration (nmol/mg)	CalcMol (ng/mg)
