# Supplementary material for: Dynamic Variation of Amino Acid Contents and Identification of Sterols in Xinyang Mao Jian Green Tea
Source: Molecules. 2022 Jun 1;27(11):3562. doi: 10.3390/molecules27113562 (PMC9182030; doi:10.3390/molecules27113562)
Supplement: Supplementary file 1 [file molecules-27-03562-s001.zip › pf-sm-chaye-yp202129670-sample-8-6ul-2021-7-3 2-13-38_REPORT-.pdf]

Hitachi Amino Acid Analyzer Report

Data File: E:\data\20210701 PF\PF-sm-chaye-yp202129670-sample-8-6ul-2021-7-3 2-13-38.dat
Method: E:\data\20210701 PF\sm-chaye.met
Sample ID: {Sample ID}
Vial Number: 158 Inj. Volume(uL): 6

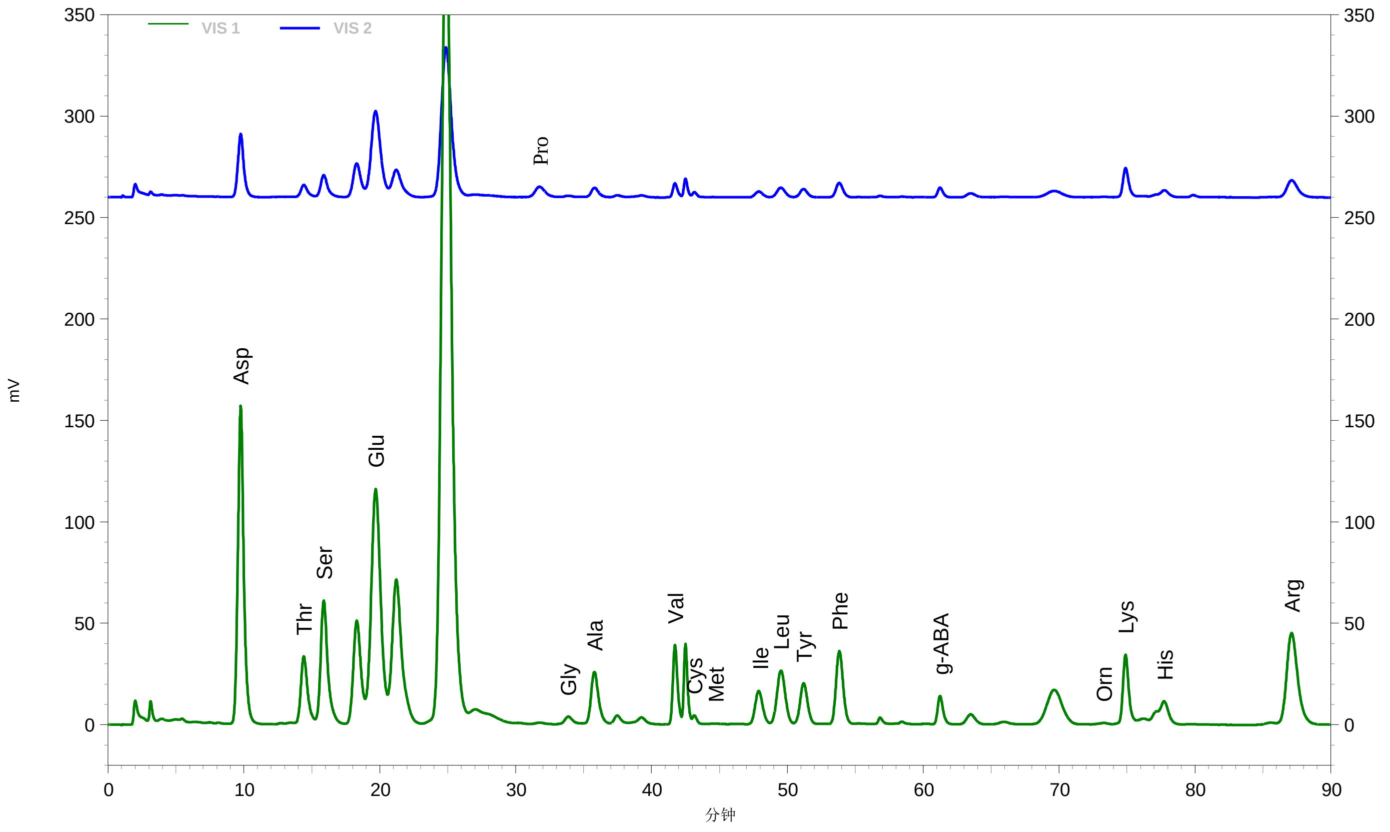

VIS 1 result

Peak #	Retention Time	Name	Area	Resolution	Mol concentration (nmol/mg)	CalcMol (ng/mg)
10	9.767	Asp	19124090	1.69	21.77	2897.93
13	14.407	Thr	4669993	0.84	5.09	606.10
14	15.873	Ser	9017937	1.61	9.78	1027.64
16	19.693	Glu	23000684	1.15	25.53	3755.79
22	33.880	Gly	628144	1.74	0.67	49.94
23	35.807	Ala	4023192	1.86	4.19	373.34
26	41.740	Val	3784026	2.66	3.80	444.92
28	43.160	Cys	371815	1.08	0.33	78.53
29	44.680	Met	30326	1.84	0.03	4.41
30	47.900	Ile	2546339	3.23	2.47	324.18
31	49.533	Leu	4497051	1.49	4.45	583.55
32	51.207	Tyr	3051402	1.54	3.04	551.50
33	53.827	Phe	5228543	2.62	5.32	878.66
40	61.240	g-ABA	1538135	1.43	1.60	164.79
45	73.293	Orn	127540	1.46	0.09	11.91
46	74.893	Lys	4224079	1.56	3.66	535.32
49	77.747	His	2033791	0.00	1.86	288.79
51	87.127	Arg	10346995	0.92	10.33	1799.38
Sum			98244082		104.00	

VIS 2 result

Peak #	Retention Time	Name	Area	Resolution	Mol concentration (nmol/mg)	CalcMol (ng/mg)
8	31.780	Pro	1006681	5.14	3.66	421.33
Sum			1006681		3.66	
