# Supplementary material for: Dynamic Variation of Amino Acid Contents and Identification of Sterols in Xinyang Mao Jian Green Tea
Source: Molecules. 2022 Jun 1;27(11):3562. doi: 10.3390/molecules27113562 (PMC9182030; doi:10.3390/molecules27113562)
Supplement: Supplementary file 1 [file molecules-27-03562-s001.zip › pf-sm-chaye-yp202129670-sample-9-6ul-2021-7-3 4-05-57_REPORT-.pdf]

Hitachi Amino Acid Analyzer Report

Data File: E:\data\20210701 PF\PF-sm-chaye-yp202129670-sample-9-6ul-2021-7-3 4-05-57.dat
Method: E:\data\20210701 PF\sm-chaye.met
Sample ID: {Sample ID}
Vial Number: 159 Inj. Volume(uL): 6

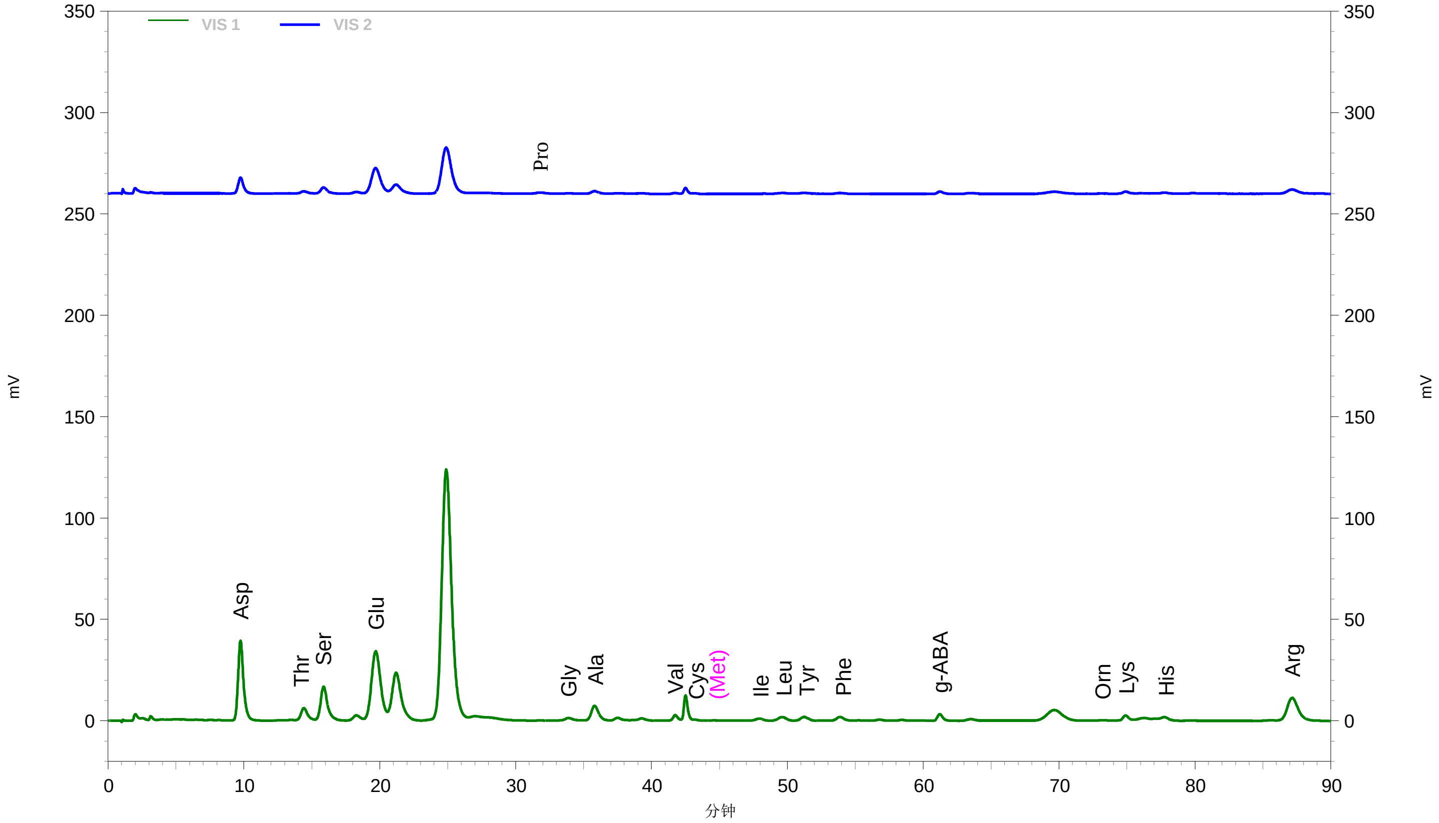

VIS 1 result

Peak #	Retention Time	Name	Area	Resolution	Mol concentration (nmol/mg)	CalcMol (ng/mg)
11	9.747	Asp	4379922	2.54	4.99	663.70
12	14.413	Thr	781788	5.94	0.85	101.47
13	15.867	Ser	2373594	1.66	2.57	270.48
15	19.700	Glu	6571597	1.23	7.29	1073.08
19	33.893	Gly	176618	2.85	0.19	14.04
20	35.813	Ala	1057077	1.95	1.10	98.09
23	41.747	Val	261764	2.79	0.26	30.78
25	43.147	Cys	43641	0.81	0.04	9.22
		Met			0.00 BDL	0.00
26	47.927	Ile	148308	4.54	0.14	18.88
27	49.633	Leu	299315	1.60	0.30	38.84
28	51.247	Tyr	286250	1.49	0.29	51.74
29	53.880	Phe	249562	2.61	0.25	41.94
32	61.233	g-ABA	358975	4.17	0.37	38.46
36	73.207	Orn	22801	2.29	0.02	2.13
37	74.900	Lys	329724	1.91	0.29	41.79
40	77.747	His	298820	0.00	0.27	42.43
41	87.160	Arg	2502199	6.82	2.50	435.14
Sum			20141955		21.72	

VIS 2 result

Peak #	Retention Time	Name	Area	Resolution	Mol concentration (nmol/mg)	CalcMol (ng/mg)
8	31.807	Pro	82147	5.65	0.30	34.38
Sum			82147		0.30	
